# Supplementary material for: Aerial survey estimates of polar bears and their tracks in the Chukchi Sea
Source: PLoS One. 2021 May 6;16(5):e0251130. doi: 10.1371/journal.pone.0251130 (PMC8101751; doi:10.1371/journal.pone.0251130)

**S1 Appendix.**  **Description of the development of polar bear resource selection layers for inclusion in model to estimate the number of polar bears in the Chukchi Sea polar bear subpopulation.**

To develop resource selection function (RSF) layers for inclusion into the model, as described in the main text of the manuscript, we used the coefficient estimates for the contemporary period published in Wilson et al. (2016). Because Wilson et al. (2016) only produced predictive RSF surfaces through 2013, we obtained updated layers for environmental covariates in the model that corresponded to each day that the instrument-based surveys occurred (i.e., 7 Apr – 31 May 2016). These updated layers included daily sea ice concentration (obtained from the National Snow and Ice Data Center; http://nsidc.org/data/nsidc-0051; accessed 11 Mar 2021) and weekly presence/absence of landfast ice (obtained from the National Snow and Ice Data Center; http://nsidc.org/data/g02172; accessed 11 Mar 2021). Each updated variable was scaled to conform with the same scaling applied in Wilson et al. (2016). That is, the same means and standard deviations for variables used in Wilson et al. (2016) were applied to the updated data for the current analysis. As in Wilson et al. (2016), habitat use predictions were based on sea ice concentration, ocean depth, standard deviation of sea ice concentration within a 100 km radius, and presence/absence of land fast ice.

Wilson et al. (2016) conducted their analysis in a Bayesian framework, so it was possible to sample from the posterior distribution of coefficient estimates to estimate the predicted RSF values at each grid cell and day used in the aerial survey analysis. We therefore obtained 2,000 posterior samples of coefficient estimates from the results of Wilson et al. (2016) and estimated the relative probability of use for each grid cell in the current analysis based on the scaled-value of the different variables (e.g., sea ice concentration) present at the cell’s centroid. We then took the median value of the 2,000 estimates for each grid cell to create the layers of polar bear RSF used in the current analysis. An RSF layer, using the above approach, was developed for each day the instrument-based surveys occurred (Fig. A1). For further details on the resource selection modeling used to obtain estimates of selection coefficients, please see the supplemental materials in Wilson et al. (2016).

**References**

Wilson, R. R., Regehr, E. V., Rode, K. D., & St Martin, M. (2016). Invariant polar bear habitat selection during a period of sea ice loss. Proceedings of the Royal Society B 283: 20160380

**Figure A1**. Depiction of the daily resource selection surfaces used in the analysis to help inform where polar bears were most likely to occur during each day of the instrument-based survey. This figure provides four examples of the daily surfaces that were used in the analysis, with the date for each map presented in the lower right corner of each panel. .


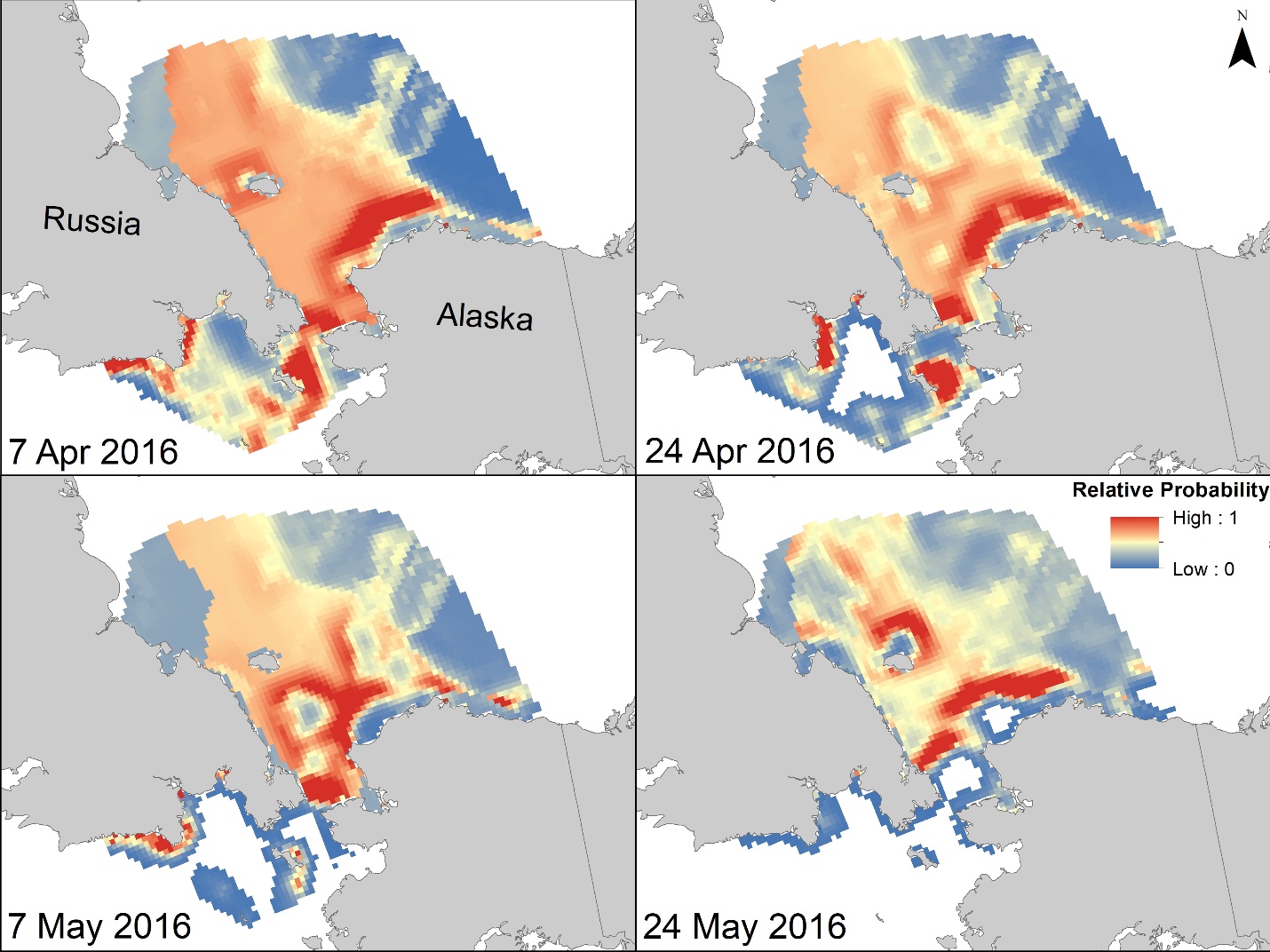

Supplement: S1 Appendix — (DOCX) [file pone.0251130.s001.docx]
