# Supplementary material for: Aerial survey estimates of polar bears and their tracks in the Chukchi Sea
Source: PLoS One. 2021 May 6;16(5):e0251130. doi: 10.1371/journal.pone.0251130 (PMC8101751; doi:10.1371/journal.pone.0251130)
Supplement: S2 Appendix — (DOCX) [file pone.0251130.s002.docx]

**S2 Appendix. Supplementary information on distance sampling.**

Here we provide additional information on distance sampling models fitted to polar bear detections in Russian surveys. Detections consisted of in situ human observations, as well as photographs made from the aircraft. In each case, perpendicular distances were recorded (for human observers, these were made using an inclinometer and aircraft altimeter data; for photographs, simple measurements were performed using known ground footprints of photographs. In many cases, bears were observed using both methods, but observations were not made independently so methods such as mark-recapture distance sampling (Borchers et al. 1998) could not be employed. A summary of distance measurements for both observation types are provided in Supplementary Figure 1.

Since observations were dependent, the only statistically coherent way to model distance measurements was to combine observation types. We first began by fitting standard distance sampling detection models to these data. In particular, we used AIC (Burnham and Anderson 2002) to compare the fits of alternative detection models to our data (Supplementary Figure 2, Supplementary Table 1). This analysis was conducted using the package “Distance” (Miller et al 2019) for R computing environment (R Development Core Team. 2016). All data were truncated to assume ½ of a transect equal to 600 m. A total of 47 sightings (both visually detected and photographed bears without double-counting) were used to construct detection functions.

Of the “standard” detection function models, the half-normal appeared to fit the detection data the best based on the lowest AIC (Supplementary Table 1). However, we were still somewhat concerned that it might not adequately characterize the detection process since distances represented a combination of observations from human observers and photographs made from the aircraft which had a fixed truncation distance that depended on the altitude of the aircraft (median 350 m). One approach would be to consider a uniform - half normal mixture model for a detection function, where the detection function is proportional to

$h\left( d \right)\propto\pi f\left( d | 0,\sigma\right)+\frac{1-\pi}{350}I_{\left[ 0,350 \right]}(d)$,

where π is a mixing proportion (treated as an unknown parameter), $f\left( d | 0,\sigma\right)$ is a half-normal probability density function with mean zero and standard deviation σ, 350 is the upper bound of the uniform distribution (set equal to the median photo width), and $I_{\left[ 0,350 \right]}(d)$ is an indicator function taking on 1.0 if distance values *d* are in [0, 350] and 0.0 otherwise. We implemented this model in R (R Development Core Team 2016), and used maximum likelihood to estimate its parameters and associated AIC score, and compared this to AIC values for a half-normal model (also implemented directly in R so that normalizing constants were comparable). The half-normal had a better AIC score than the mixture model (ΔAIC = 1.6), so we opted to use the half-normal model to analyze Russian polar bear detection data.

We also compiled a list of g(0) (proportion of bears detected on the transect line) values from the literature in hopes that they might guide our choice for g(0) for the polar bear model (Supplementary Table 2). Unfortunately, all surveys in the literature were conducted at substantially lower altitudes and at slower speeds than we flew in our surveys which makes selection of a g(0) values difficult.


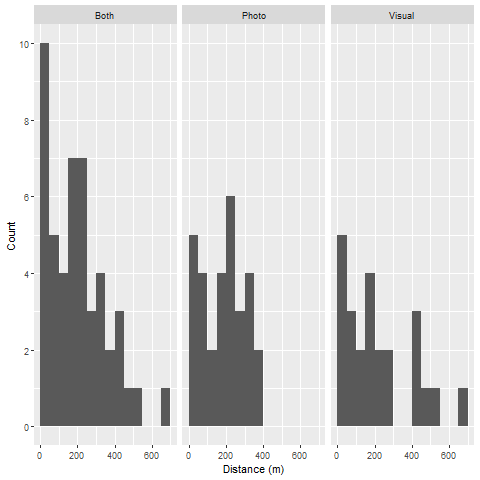


Supplementary Figure 1. Number of observations by perpendicular distance from the transect line (in 50 m bins) as a function of how data were gathered. Distance bins for photographs (“Photo”) were restricted based on the camera visual footprint (itself a function of altitude), while visual observations (“Visual”) could be made to greater distances. The “Both” category indicates observations made by either method (including bears detected by both methods). Note that these plots do not include one bear observed at ~1200 m, which was censored prior to analysis.


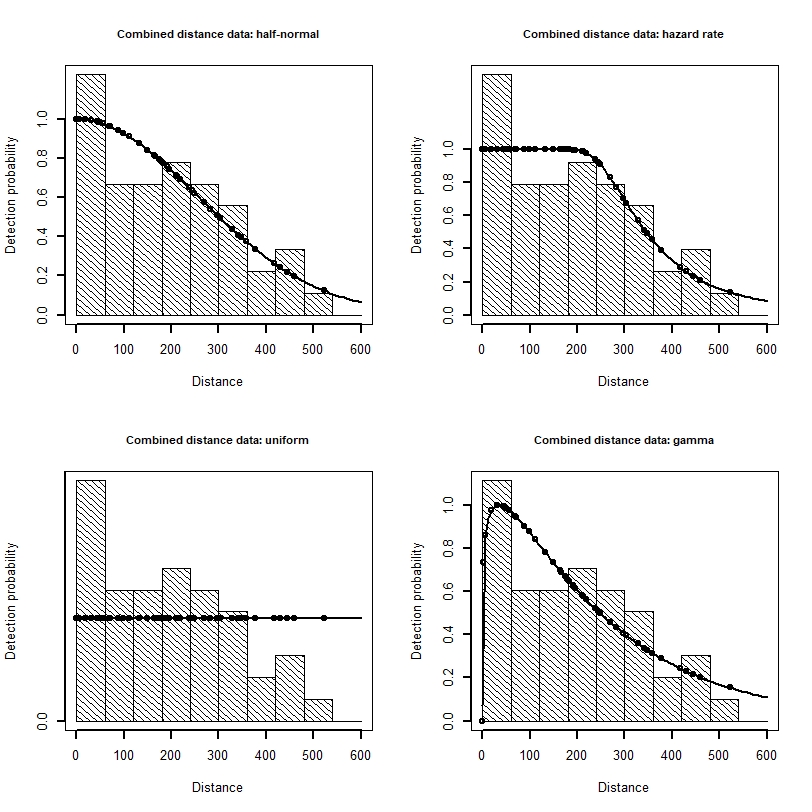


Supplementary Figure 2. Detection functions for combined (visual and photo detections) data from polar bear aerial survey

**Supplementary Table 1 – Detection function comparison results**

| Detection function | Scale coefficient (SE) | Average Pr (SE)  (non-corrected for g(0)<1) | AIC |
| --- | --- | --- | --- |
| Half-normal | 5.54 (0.145) | 0.52 (0.067) | 583.79 |
| *- cosine* |  |  | *585.55* |
| *- Hermite* |  |  | *584.75* |
| *- simple polynomial* |  |  | *584.77* |
| Hazard-rate | 1.33 (0.534) | 0.62 (0.071) | 586.05 |
| Uniform |  | 0.002 | 601.31 |
| Gamma | -1.82 (1.499) | 0.48 (0.085) | 643.81 |

**Supplementary Table 2 - g(0) estimates from published polar bear visual survey results.**

| nn | Author | Survey region | Period | Substrate | Platform | Speed | Altitude | g(0) | Comment |
| --- | --- | --- | --- | --- | --- | --- | --- | --- | --- |
| 1 | Aars et al 2017 | Svalbard | 30 Jul - 15 Aug | Land and sea ice | Helicopter Eurocopter AS350 Ecureuil | 185  km/h | 200 ft (61 m) | Assumed 1.0 | ". . . g(0) likely was very close to 1 on land, close to 1 in areas with flat sea ice; “. . . the areas with very screwed sea ice where g(0) could have been considerably lower than 1 only constituted a very minor part of the total survey area ". |
| 2 | Stapleton et al 2016 | Foxe Basin, N Quebec | Aug–Sep 2009 and Aug–Oct 2010 | Land | Helicopter Bell 206 LongRanger | 150 km/h | 400 ft (120 m) | 0.97-0.98 | "This is a probability of a bear located 75 m from the aircraft being sighted by at least one observer. This finding suggests that the assumption of complete detection at distance 0 was approximately valid, consistent with our perception in the field". |
| 3 | Aars et al 2009 | Barents Sea | 26 Jul - 1 Sep 2004 | Land and sea ice | Helicopter Eurocopter AS350 Ecureuil | 185 km/h | 200 ft (61 m) | Assumed 1.0 | "… along transects covering these habitats, g(0) was either 1 or very close to 1. However, in some sections of transects . . . with heavily packed ice, it is possible that polar bears close to the line could be missed”; “. . . Even if g(0) was considerably lower than 1 in structure 3 habitat, we think that g(0) was close to 1 when averaged over all transects". |
| 4 | Obbard et al 2015 | S Hudson Bay | Late summer, ice free season | Land | Helicopter Eurocopter EC-130 | 160 km/h | 400 ft (120 m) | Pr_front_: 0.86 (SE = 0.07); Pr_rear_: 0.63 (SE = 0.08),  Pr_overall_ = 0.95 | Probability for front and rear observers and for both observers together to see a bear |
| 5 | Dyck et al 2017 | W Hudson Bay | Summer | Land | Twin Otter fixed wing; Eurocopter EC135 twin engine rotary wing aircraft; Bell L4 and LR4 Long Ranger | 160 km/h | 400 ft (120 m) | Pr_fornt_ = 0.63; Pr_rear_: = 0.76; Pr_overall_ = 0.9 | Combined double observer prob =0.9; Average detection probabilities for the front and rear observer was 0.63 and 0.76 |
| 6 | Evans et al 2003 | E Chukchi and W Beaufort | Aug | Sea ice | USCG HH-65A helicopters Aerospatiale Dauphine AS 365N2 | 145 km/h | 300 ft (91 m) | 0.67 (SE = 0.144) | "We assumed that sightability during the 2000 survey was similar to the sightability from either the front or the back seat of the 1994 (McDonald et al., 1999), survey and that sightability in both surveys was the same on the left and right sides of the aircraft". |
| 7 | McDonald et al 1999 | N Alaska | 9 Apr - 2 May 1987 | Sea ice | Twin Otter fixed wing | 222 km/h | 300 ft (91 m) | Assumed 1.0 | [note: "distance correction factor for the focal strip" estimated but not the same thing as g(0)] |
| 8 | McDonald et al 1999 | Beaufort Sea | 6-19 Jun 1994 | Sea ice | Helicopter Bell 212, Twin otter fixed wing | 185 km/h | 300 ft (91 m) | 0.88 | Pr(At least one of 2 observers sees a bear) - helicopter only. In the fixed wing, 6 bears were detected by front observer, 1 by back observer, and neither saw the same groups of bears |
| 9 | Wiig, Derocher 1999 | Barents Sea | 28 Feb - 7 Mar 1987 | Sea ice | Helicopter Lynx | 160 km/h | 200 ft (61 m) | Assumed 1.0 | Not estimated |

**References**

Aars J., Marques T.A., Lone K., Andersen M., Wiig Ø., Bardalen Fløystad I.M., Hagen S.B.&

Buckland S.T. 2017. The number and distribution of polar bears in the western Barents Sea, Polar Research, 36:1, DOI: 10.1080/17518369.2017.1374125

Aars J., Marques T.A., Buckland S.T., Andersen M., Belikov S., Boltunov A. & Wiig Ø. 2009. Esti

mating the Barents Sea polar bear subpopulation size. Marine Mammal Science 25, 35–52.

Borchers, D.L., Zucchini, W. & Fewster, R.M. 1998. Mark‐recapture models for line transect surveys. Biometrics 54: 1207–1220.

Dyck M., Campbell M., Lee D.S., Boulanger J., and Hedman D. 2017. Aerial survey of the western

Hudson Bay polar bear sub-population 2016. 2017 Final Report. Government of Nunavut, Department of Environment, Wildlife Research Section, Status Report 2017-xx, Igloolik, NU. 82 pp + 2 Supplements.

Evans T., Fischbach A., Schlieb S., Manly B., Kalxdorff S., & York G. 2003. Polar Bear Aerial Survey

in the Eastern Chukchi Sea: A Pilot Study. Arctic, 56(4), 359-366. Retrieved from www.jstor.org/stable/40513075

McDonald L. & Garner G. & Robertson, D. 1999. Comparison of aerial survey procedures for

estimating polar bear density: Results of pilot studies in Northern Alaska.

Miller D.L., Rexstad E., Thomas L., Marshall L., Laake J.L. 2019. “Distance Sampling in R.” Journal

of Statistical Software, *89*(1), 1-28. doi: 10.18637/jss.v089.i01 (URL: https://doi.org/10.18637/jss.v089.i01).

Obbard M.E., Stapleton S., Middel K.R., Thibault I., Brodeur V. & Jutras C. 2015. Estimating the

abundance of the Southern Hudson Bay polar bear subpopulation with aerial surveys. Polar Biology 38, 1713–1725.

R Development Core Team. R: A Language and Environment for Statistical Computing. R

Foundation for Statistical Computing, Vienna, Austria (2016). URLhttp://www.R-project.org. ISBN 3-900051-07-0

Stapleton S., Peacock E. & Garshelis D. 2016. Aerial surveys suggest long-term stability in the

seasonally ice-free Foxe Basin (Nunavut) polar bear population. Marine Mammal Science 32, 181–201.

Wiig Ø, and Derocher A.E. 1999. Application of aerial survey methods to polar bears in the Barents

Sea: Marine Mammal Survey and Assessment Methods, A.A. Balkema, 3000 BR Rotterdam Netherlands, pp. 27-36.
