# Supplementary material for: Aerial survey estimates of polar bears and their tracks in the Chukchi Sea
Source: PLoS One. 2021 May 6;16(5):e0251130. doi: 10.1371/journal.pone.0251130 (PMC8101751; doi:10.1371/journal.pone.0251130)

PLOS ONE

Aerial survey estimates of polar bears and their tracks in the Chukchi Sea

--Manuscript Draft--

**Manuscript Number:**	PONE-D-20-23811
**Article Type:**	Research Article
**Full Title:**	Aerial survey estimates of polar bears and their tracks in the Chukchi Sea
**Short Title:**	Chukchi Sea polar bear abundance
**Corresponding Author:**	Paul B Conn<br>National Marine Fisheries Service<br>Seattle, Washington UNITED STATES
**Keywords:**	Aerial survey; polar bears; species distribution model; thermal imagery; Ursus maritimus
**Abstract:**	Polar bears are of international conservation concern due to climate change but are difficult to study because of large ranges and low densities. In a collaborative American-Russian effort in spring of 2016, we used aerial surveys to detect and estimate the abundance of polar bears on sea ice in the Chukchi Sea. Our surveys used a combination of thermal imagery, digital photography, and human observations. Using spatio-temporal statistical models that related bear and track densities to physiographic and biological covariates (e.g., sea ice extent, resource selection functions derived from satellite tags), we predicted abundance and spatial distribution throughout our study area. Estimates of 2016 abundance ([[EQUATION]]) ranged from 3,555 (95% CI: 2,382-5,305) to 5,623 (95% CI: 3,761-8,408) depending on the proportion of bears assumed to be missed on the transect line during Russian surveys (g(0)). Our estimates are larger than, but of similar magnitude to, a recent mean estimate for the period 2008-2016 ([[EQUATION]] = 2,937; 95% CI 1,522-5,944) derived from an integrated population model applied to a slightly smaller area. Although the upper bound of population size is extremely imprecise because of unknown g(0) values, it establishes a useful lower bound for abundance, and suggests high spring polar bear densities on sea ice in Russian waters. Though imprecise, we suggest that springtime aerial surveys may represent a plausible avenue for studying abundance and distribution of polar bears over large, remote areas. We provide several suggestions that should help improve future aerial surveys.
**Order of Authors:**	Paul B Conn
	Vladimir Chernook
	Erin Moreland
	Irina Trukhanova
	Eric Regehr
	Alexander Vasiliev
	Ryan Wilson
	Stansislav Belikov
	Peter Boveng
**Additional Information:**	
**Question**	**Response**
**Financial Disclosure**<br><br>Enter a financial disclosure statement that describes the sources of funding for the work included in this submission. Review the submission guidelines for detailed	Funding for surveys was provided primarily by the U.S. National Oceanic and Atmospheric Administration (NOAA) and the U.S. Fish & Wildlife Service (USFWS). Support for Russian surveys was provided by NOAA through the North Pacific Wildlife Consulting, LLC (http://www.northpacificwildlife.com/). Portions of the analysis were supported by joint subaward NA17NMF4720289, project 1813, from the North Pacific Research Board and The Prince William Sound Oil Spill Recovery Institute

Powered by Editorial Manager® and ProduXion Manager® from Aries Systems Corporation

requirements. View published research articles from *PLOS ONE* for specific examples.<br><br>This statement is required for submission and **will appear in the published article** if the submission is accepted. Please make sure it is accurate.<br><br>**Unfunded studies**<br>Enter: *The author(s) received no specific funding for this work.*<br><br>**Funded studies**<br>Enter a statement with the following details:<br>• Initials of the authors who received each award<br>• Grant numbers awarded to each author<br>• The full name of each funder<br>• URL of each funder website<br>• Did the sponsors or funders play any role in the study design, data collection and analysis, decision to publish, or preparation of the manuscript?<br>• **NO** - Include this sentence at the end of your statement: *The funders had no role in study design, data collection and analysis, decision to publish, or preparation of the manuscript.*<br>• **YES** - Specify the role(s) played.<br><br>* typeset	(https://www.nprb.org/core-program/about-the-program/; PL, ER, IT, EM, and PC were principal or co-investigators). Additional support for data processing and survey logistics on the Russian side was provided by USFWS, the RPO Marine Mammal Council (https://marmam.ru/en/) and WWF Russia (https://wwf.ru/en/about/) in funding agreements with VC. External funders (e.g., NPRB, WWF Russia) had no role in study design, data collection and analysis, decision to publish, or preparation of the manuscript.
**Competing Interests**<br><br>Use the instructions below to enter a competing interest statement for this submission. On behalf of all authors, disclose any competing interests that could be perceived to bias this work—acknowledging all financial support and any other relevant financial or non-financial competing interests.<br><br>This statement **will appear in the published article** if the submission is accepted. Please make sure it is accurate. View published research articles from *PLOS ONE* for specific examples.	The authors have declared that no competing interests exist

**NO authors have competing interests**<br><br>Enter: *The authors have declared that no competing interests exist.*<br><br>**Authors with competing interests**<br><br>Enter competing interest details beginning with this statement:<br><br>*I have read the journal's policy and the authors of this manuscript have the following competing interests: [insert competing interests here]*<br><br>* typeset	
**Ethics Statement**<br><br>Enter an ethics statement for this submission. This statement is required if the study involved:<br><br>• Human participants<br>• Human specimens or tissue<br>• Vertebrate animals or cephalopods<br>• Vertebrate embryos or tissues<br>• Field research<br><br>Write "N/A" if the submission does not require an ethics statement.<br><br>General guidance is provided below. Consult the submission guidelines for detailed instructions. **Make sure that all information entered here is included in the Methods section of the manuscript.**	Aerial surveys were conducted under National Marine Fisheries Service permit Permit No. 19309 and U.S. Fish & Wildlife Service Permit No. MA212570-1

Powered by Editorial Manager® and ProduXion Manager® from Aries Systems Corporation

**Format for specific study types**<br><br>**Human Subject Research (involving human participants and/or tissue)**<br>• Give the name of the institutional review board or ethics committee that approved the study<br>• Include the approval number and/or a statement indicating approval of this research<br>• Indicate the form of consent obtained (written/oral) or the reason that consent was not obtained (e.g. the data were analyzed anonymously)<br><br>**Animal Research (involving vertebrate animals, embryos or tissues)**<br>• Provide the name of the Institutional Animal Care and Use Committee (IACUC) or other relevant ethics board that reviewed the study protocol, and indicate whether they approved this research or granted a formal waiver of ethical approval<br>• Include an approval number if one was obtained<br>• If the study involved *non-human primates*, add *additional details* about animal welfare and steps taken to ameliorate suffering<br>• If anesthesia, euthanasia, or any kind of animal sacrifice is part of the study, include briefly which substances and/or methods were applied<br><br>**Field Research**<br><br>Include the following details if this study involves the collection of plant, animal, or other materials from a natural setting:<br>• Field permit number<br>• Name of the institution or relevant body that granted permission	
**Data Availability**<br><br>Authors are required to make all data underlying the findings described fully available, without restriction, and from the time of publication. PLOS allows rare exceptions to address legal and ethical concerns. See the PLOS Data Policy and FAQ for detailed information.	Yes - all data are fully available without restriction

A Data Availability Statement describing where the data can be found is required at submission. Your answers to this question constitute the Data Availability Statement and **will be published in the article**, if accepted.<br><br>**Important:** Stating 'data available on request from the author' is not sufficient. If your data are only available upon request, select 'No' for the first question and explain your exceptional situation in the text box.<br><br>Do the authors confirm that all data underlying the findings described in their manuscript are fully available without restriction?	
**Describe where the data may be found in full sentences. If you are copying our sample text, replace any instances of XXX with the appropriate details.**<br><br>• If the data are **held or will be held in a public repository**, include URLs, accession numbers or DOIs. If this information will only be available after acceptance, indicate this by ticking the box below. For example: *All XXX files are available from the XXX database (accession number(s) XXX, XXX.).*<br>• If the data are all contained **within the manuscript and/or Supporting Information files**, enter the following: *All relevant data are within the manuscript and its Supporting Information files.*<br>• If neither of these applies but you are able to provide **details of access elsewhere**, with or without limitations, please do so. For example:<br><br>*Data cannot be shared publicly because of [XXX]. Data are available from the XXX Institutional Data Access / Ethics Committee (contact via XXX) for researchers who meet the criteria for access to confidential data.*<br><br>*The data underlying the results presented in the study are available from (include the name of the third party*	Code and data to recreate analyses is currently located at https://github.com/pconn/ChukchiPolarBear and will be permanently archived to a public repository (e.g. figshare, zenodo) following manuscript acceptance

and contact information or URL).<br>• This text is appropriate if the data are owned by a third party and authors do not have permission to share the data.<br><br>* typeset	
Additional data availability information:	Tick here if the URLs/accession numbers/DOIs will be available only after acceptance of the manuscript for publication so that we can ensure their inclusion before publication.

Powered by Editorial Manager® and ProduXion Manager® from Aries Systems Corporation

Manuscript Click here to access/download;Manuscript;ConnEtAl_ms.pdf

Aerial survey estimates of polar bears and their tracks in the Chukchi Sea

Paul B. Conn [1*], Vladimir I. Chernook [2], Erin E. Moreland [1], Irina S. Trukhanova [3], Eric V. Regehr [4,5], Alexander N. Vasiliev [2], Ryan R. Wilson [4], Stanislav E. Belikov [6], Peter L. Boveng [1]

1 Alaska Fisheries Science Center, National Marine Fisheries Service, NOAA, 7600 Sand Point Way NE, Seattle, WA 98115, USA

2 Autonomous Non-Commercial Organization, Ecological Center, ECOFACTOR, 11/1-10N Neyshlotskiy Lane, Saint-Petersburg, 194044 Russia

3 North Pacific Wildlife Consulting, LLC, 12600 Elmore Road, Anchorage, AK 99516, USA

4 US Fish and Wildlife Service, Marine Mammals Management, 1011 East Tudor Road, Anchorage, AK 99503, USA

5 Polar Science Center, Applied Physics Laboratory, University of Washington, 1013 NE 40th St, Box 355640, Seattle, WA, USA

6 FSBI "All-Russian Research Institute for Nature Protection" 36km MKAD, 1-4, Moscow, 117628, Russia

* paul.conn@noaa.gov

Abstract

Polar bears are of international conservation concern due to climate change but are difficult to study because of large ranges and low densities. In a collaborative American-Russian effort in spring of 2016, we used aerial surveys to detect and estimate the abundance of polar bears on sea ice in the Chukchi Sea. Our surveys used a combination of thermal imagery, digital photography, and human observations. Using spatio-temporal statistical models that related bear and track densities to physiographic

and biological covariates (e.g., sea ice extent, resource selection functions derived from satellite tags), we predicted abundance and spatial distribution throughout our study area. Estimates of 2016 abundance ($\hat{N}^*$) ranged from 3,555 (95% CI: 2,382-5,305) to 5,623 (95% CI: 3,761-8,408) depending on the proportion of bears assumed to be missed on the transect line during Russian surveys ($g(0)$). Our estimates are larger than, but of similar magnitude to, a recent mean estimate for the period 2008-2016 ($\hat{N}^* = 2,937$; 95% CI 1,522-5,944) derived from an integrated population model applied to a slightly smaller area. Although the upper bound of population size is extremely imprecise because of unknown $g(0)$ values, it establishes a useful lower bound for abundance, and suggests high spring polar bear densities on sea ice in Russian waters. Though imprecise, we suggest that springtime aerial surveys may represent a plausible avenue for studying abundance and distribution of polar bears over large, remote areas. We provide several suggestions that should help improve future aerial surveys.

Introduction

Polar bears are distributed throughout the circumpolar Arctic in 19 partially discrete subpopulations [1]. In 2008, the species was listed as threatened under the U.S. Endangered Species Act due to loss of sea-ice habitat resulting from climate change [2]. Currently, the demographic status of the 19 subpopulations is variable due to geographic variation in sea-ice conditions, ecosystem function, and other factors [3, 4]. Subpopulation-specific data are required to understand the effects of climate change and inform localized conservation solutions, including management of subsistence harvests that provide nutritional, cultural, and economic benefits to indigenous people [5, 6]. However, accurate and timely population data are difficult and expensive to collect because polar bears inhabit remote regions in low densities (e.g., 0.0041 bears/km[illegible]) [7].

The Chukchi Sea (CS) polar bear subpopulation (also referred to as the Alaska-Chukotka population, with different boundaries) ranges widely on sea ice of the northern Bering, Chukchi, and East Siberian seas [8] and is managed under a bilateral treaty between the U.S. and Russia [1]. The first estimates of Alaska-Chukotka polar bear abundance (2000-5000 bears) were published in the early 1990s, based on both expert opinion and the number of maternal dens counted on Wrangel Island, Russia

(inflated to account for breeding females comprising 8-10% of the population) [9, 10]. Live-capture research in the U.S. between 2008 and 2016 suggested that CS bears displayed good nutritional condition [11, 12] and productive demography [13] despite sea-ice loss. An integrated population model fitted to these data and extrapolated to the CS region produced an estimate of 2,937 (95% CI 1522-5944) bears. Capture research is unlikely to be performed annually, however, because of high costs and human safety concerns due to progressive deterioration of spring sea-ice conditions. Alternative study methods are needed to understand the mechanisms through which sea-ice loss is affecting CS bears and to periodically update population data according to a harvest management framework adopted in 2018 [14].

One possible approach for surveying polar bear subpopulations is to use springtime, instrument-based aerial surveys. These surveys use infrared cameras and coordinated digital color photography to detect the warm bodies of animals on sea ice and confirm species identity. Such surveys have proven extremely effective for estimating ice-associated pinniped abundance [15–17], but have yet to be applied to polar bears (except to detect dens [18]).

To better understand the distribution and abundance of seals and polar bears in the CS region, we conducted a comprehensive, instrument-based aerial survey in April and May of 2016. Although our survey generated data on multiple species, here we focus on polar bears, including direct observations of animals and observations of tracks in snow accumulated on sea ice. We provide an overview of the survey area and study design, including technical specifications of survey equipment and data processing protocols, and describe novel statistical methods. We then use our methods to estimate the distribution and abundance of polar bears in the CS region.

Materials and methods

Study area

We conducted aerial surveys over the Chukchi Sea between April 7 and May 31, 2016. Our study area was bounded to the south by the Bering Strait, to the north by the U.S. and Russian Exclusive Economic Zone boundaries, to the west by the 156° W line, and

to the east by a line extending north from Chaunskaya Bay, Russia (Fig 1). We divided our study area into grid cells that were approximately 25 km by 25 km, based on a variant of the Lambert azimuthal equal-area projection. Removing portions of grid cells that included land, the total area of our study area was 798,049 km^2.

Fig 1. Chukchi Sea study area. A map of the study area used in 2016 aerial surveys for seals and polar bears. Black lines represent aerial survey tracks, while small blue circles represent locations of bear tracks. Red triangles represent thermal detections of polar bear groups, and orange squares represent additional groups seen by human observers (U.S.) and human observers or in post hoc examination of photographs (Russia). The $\approx 625\text{km}^2$ grid cells used for abundance estimation appear in the background (beige lines). Land masses (gray) include Alaska, U.S.A. to the east and Russia, to the west. The blue shading represents the area associated with the Chukchi subpopulation of polar bears as determined by the polar bear specialist working group (PBSG), while yellow shading shows the area where a long term capture-recapture study of polar bears [13] was previously conducted.

Aerial survey platform and protocols

We used two aircraft with different observation platforms to conduct aerial surveys. In U.S. airspace, surveys were flown in a King Air A90, which was configured with three downward facing, cooled LWIR cameras (FLIR A6750sc SLS) with 25 mm lenses mounted in the bellyport. An uncooled LWIR microbolometer (FLIR A645sc) was used as a backup camera for a portion of the survey. The thermal cameras had a viewing angle of 24.9° and temperature sensitivity of 0.03° C. Each of these thermal cameras was paired with a machine vision 29 megapixel camera with a 100 mm lens which provided matching color images. Camera pairs were mounted in an array with the center pair in a nadir position, and side pairs mounted at 25.5° angles inward, providing an overall field of view of 76° for the thermal cameras. Flying at a target altitude of 305 m, the ground resolution of the thermal cameras was 20-23 cm/pixel with a swath width of 470 m. The ground resolution of the color cameras was 1.71-2.13 cm/pixel with a footprint that closely matched (but did not completely cover) the thermal footprint of each camera. Image collection between the color and thermal cameras was synchronized and images were collected continuously at a rate of approximately 2 fps, providing forward moving overlap of images when flying at 120-140 kts. In addition to collecting automated color and thermal imagery, the U.S. survey team recorded visual observations of bears that occurred outside the range of the thermal swath.

In Russia, surveys were flown using an AN-26 Arctica aircraft equipped with a Malachite-M infrared (IR) scanner with a wide viewing angle of 88° and temperature sensitivity of 0.2° C. An Optris PI 450 thermal camera was used as a backup. Flying at an average speed of 160 kts and a target altitude of 250 m, the IR ground footprint was 483 m and was completely covered by three digital color 36 megapixel cameras with 50 mm lenses. The IR-scanner resolution at the target altitude was ≈11 cm/pixel and the resolution of color cameras was 2.27 cm/pixel. Color cameras could be triggered by any of the following events: 1) IR-scanner detected a hot spot on ice; 2) IR-scanner operator (a technician monitoring the IR-video stream in real time) detected an anomaly (possible hot spot, not strong enough to be picked up by the detection algorithm) on the screen and hit the "shoot" button; or 3) one of the visual observers saw an object of interest on ice (e.g., animal, tracks, kill site, water access hole) and hit the "shoot" button. Additionally, the color cameras made photographs at a set interval of 20 sec (for May flights only). In addition to imagery, four visual observers (two on each side of the aircraft) made continuous observations through bubble windows and used hand-held inclinometers to determine the angle at which an object of interest was observed in order to estimate detection distance.

U.S. and Russian survey protocols differed substantially, mostly owing to the constraints imposed by the survey platforms used. To avoid potential for bias due to preferential sampling [19, 20] crews of both aircraft were instructed to avoid fine scale targeting of ice habitat when making and altering flight plans as sea ice conditions changed, and to spread effort out over time and space as common in space-filling designs [21] in spatial statistics.

Data and data processing

Hot spot detection & post hoc searching of photographs

After surveys were completed, U.S. analysts used a general outlier algorithm to detect warm heat signatures of animals on sea ice and matched infrared detections with paired color photographs. Due to high false-positive rates of the algorithm, manual review of detections was implemented to eliminate hot spots caused by features other than animals (e.g., melt pools, dirty ice). Since the infrared scanner on Russian survey tracks

appeared not to detect many bears, Russian analysts opted to manually examine each color photograph for presence of polar bears. For each bear detected in Russian surveys, analysts calculated the distance of the bear from the transect line using standard trigonometry.

Polar bear tracks

Polar bear tracks were frequently encountered in our surveys, and we summarized the frequency of polar bear tracks as a potential explanatory variable for spatial variation in polar bear density. For U.S. flights, we reviewed every 20th color image from our port-side camera and recorded whether or not it included tracks. In Russian flights, we summarized the number of tracks recorded by human observers in each surveyed grid cell.

Polar bear detection trials

Previous studies of ice-associated seals documented high (e.g., $p = 0.96$) detection probabilities using the same thermal detection algorithm applied to this data set [17]. However, aerial thermal imagery was not available to evaluate the accuracy of the algorithm for polar bears prior to this survey effort. Despite the high emissivity of polar bear hair [22], the thermal signature of bears was likely to differ from that of seals based on the texture of the bears fur and the shape of the animal [23]. To determine the detection probability of polar bears in U.S. survey flights, we conducted experimental flyovers. Specifically, each time a polar bear was detected from the air by a human observer, we continued on transect past the bear and then went "off effort" and attempted focused fly overs while recording thermal imagery and digital photography. We also conducted flights over polar bears congregating near whale carcasses close to the village of Utqiaġvik (formerly, Barrow), Alaska. In each case, a technician manually reviewed digital photographs with time stamps associated with our fly-overs in an effort to locate polar bears. Such polar bear detections became trials with which to estimate detection probability of our thermal detection algorithm. In total, we documented 12 unique polar bears in flyovers and detected 8 with our thermal detection algorithm and manual hot spot review, for an apparent detection probability of $\hat{p}_{us} = 0.67$.

The Russian aircraft was considerably less maneuverable than the U.S. King Air so

focused flyovers were not tenable. Further, the Malechite-M thermal scanner appeared to have trouble detecting bears. No bears were detected in temperatures $< -5°C$ (temperatures typical of April survey flights). Even when temperatures were $> -5°C$, the thermal scanner detected just 1 out of 9 visually detected bears. Due to the unreliability of the Russian thermal sensor, we relied on distance sampling to estimate detection probability (see *Polar bear counts*, below).

Explanatory covariates

We assembled a number of environmental and landscape covariates that we thought might help to predict the density of polar bears and their tracks, including

- `dist_land`: distance from the centroid of each grid cell to the closest point of land (Alaska or Russia);
- `RSF`: an estimate of the relative density of space use obtained by fitting resource selection functions (RSFs) to data from fixes from satellite-collared female polar bears. We followed the same procedure as Wilson et al. (2016) [8], updating their RSFs with environmental data from 2016.
- `ice`: daily remotely sensed sea ice concentration values, as obtained from the National Snow and Ice Data Center, Boulder, Colorado, USA;
- `easting`: standardized longitude-like values in projected geographic space;
- `northing`: standardized latitude-like values in projected geographic space;
- `Water99`: a binary indicator for whether or not concentrations were less than 1%.

The `ice` covariate was often missing or unreliable in grid cells that bordered mainland Alaska or Russia. For these cells, values of `ice` were interpolated via kriging using the `autoKrige` function in the `automap` library [24] within the R programming environment [25]. Absolute correlations between explanatory covariates were all < 0.5, with exception of `northing` and `dist_land`, which had a correlation of 0.75.

Statistical analysis

We fitted several spatio-temporal statistical models to polar bear track and count data. In each case, we modeled track and count data simultaneously and aggregated them

into discrete grid cells $\approx 25 \times 25$ km^2 (Fig 1) prior to analysis.

Polar bear tracks

In order to derive a single covariate describing density of polar bear tracks, we jointly modeled U.S. and Russian track data. This was challenging since these data were summarized on different scales, requiring a common currency to describe both binary detection data in photographs (U.S.) and total number of tracks observed by human observers (Russia). We start by making the simplifying assumption that the distribution of tracks are governed by a “blocky” spatial Poisson point process with rate parameter $Z_{s,t}$ that is assumed constant for surveys of grid cell s at time t. According to this model, the probability of observing $Y = y$ tracks in a an arbitrary area B is

$$\Pr(Y = y) = \frac{(BZ_{s,t})^y}{y!} \exp(-BZ_{s,t}).$$

Technically this model assumes spatial independence of tracks, which is surely not satisfied since tracks are often continuous circuitous features left by a relatively small number of animals. However, we were willing to accept some level of model misspecification and attendant overdispersion in order to come up with a joint predictive covariate, especially since we were not as interested in absolute track density or accurate estimation of its variance.

Using this formulation for track density, and setting $B = 0.012$ km^2 (the average ground footprint of an examined photograph), the probability of observing one or more tracks in a U.S. photograph is

$$\phi_{s,t} = \Pr(Y > 0) = 1 - \exp(-0.012 Z_{s,t}).$$

We then modeled the number of U.S. photographs with polar bear tracks in cell s at time t ($T_{s,t,us}$) as

$$T_{s,t,us} | P_{s,t} \sim \text{Binomial}(T_{s,t,us}; P_{s,t}, \phi_{s,t}),$$

where $P_{s,t}$ gives the number of photographs examined in cell s at time t.

For Russian track detections, we modeled the number of tracks recorded by human

observers in cell s at time t ($T_{s,t,rus}$) as

$$T_{s,t,rus} \sim \text{Poisson}(L_{s,t}\eta Z_{s,t}), \qquad (1)$$

where $L_{s,t}$ gives the length (km) of the transect flown over cell s at time t. We included an additional scaling parameter, η, in Eq (1) because (i) there was uncertainty about the effective strip width for tracks since distances were not recorded, and (ii) we were uncertain about how separate tracks were delineated. For instance, it might be possible for a single set of polar bear tracks to encompass a large area. This scaling parameter thus helps to spatially align U.S. and Russian datasets.

We imparted spatio-temporal variation in $Z_{s,t}$ using the construction

$$\log(Z_{s,t}) = \mathbf{x}'_{s,t}\boldsymbol{\beta},$$

where $\mathbf{x}_{s,t}$ is a column vector of predictive covariates for cell s at time t and $\boldsymbol{\beta}$ is vector of regression coefficients. For details on specific models fit, see *Models and model fitting.*

Polar bear counts

When the goal of a survey is to estimate absolute abundance, several avenues exist for modeling spatio-temporal variation in animal counts [26]. However, when data are sparse (as in the case of polar bear counts), considerable stability is introduced by assuming a population closure assumption, whereby abundance is assumed constant in a study area but animals are allowed to redistribute themselves as conditions change (e.g., as ice melts) [26, 27].

We adopted such a dynamic redistribution model for polar bear abundance, but focus on the number of distinct polar bear groups (typically 1-3 bears) and employ a separate model for group size (see below). In particular, we assumed a fixed number of N polar bear groups were present in the study area on each day of the survey, and that the number of bear groups in each grid cell s ($s = 1, 2, \ldots, S$) at time t ($t = 1, 2, \ldots, T$), $N_{s,t}$, could be described as

$$N_{s,t} = N\pi_{s,t}.$$

Here, $\pi_{s,t}$ gives the probability a polar bear group is in grid cell s at time t (note that

$\sum_s \pi_{s,t} = 1.0$). We specify a multinomial link function for π_{st}, such that

$$\pi_{s,t} = A_s \exp(\nu_{s,t}) / \sum_s A_s \exp \nu_{s,t},$$

where $\nu_{s,t}$ represents a linear predictor (without an intercept), and A_s denotes the proportion of grid cell s that is composed of saltwater habitat. Predictive covariates are introduced within a linear modeling construct, such that

$$\nu_{s,t} = \mathbf{y}'_{s,t}\boldsymbol{\alpha},$$

where $\mathbf{y}_{s,t}$ is a vector of predictive covariates for grid cell s at time t and $\boldsymbol{\alpha}$ is a vector of regression parameters.

We modeled polar bear group counts with a Poisson distribution, such that

$$C_{s,t}|N_{s,t}, p_{s,t} \sim \text{Poisson}(N_{s,t}p_{s,t}).$$

where $p_{s,t} = \theta_{s,t}a_{s,t}$ is a compound detection probability that represents a number of components, including (i) imperfect detection of bears on ice ($\theta_{s,t}$), and (ii) incomplete coverage of aerial surveys over grid cell s, such that $a_{s,t}$ represents the proportion of salt water habitat in grid cell s that is surveyed during transects on day t. For U.S. surveys, we limited thermal detections to those where species were confirmed on photographs, so we calculated $a_{s,t}$ using the total ground footprint of photographs. For Russian surveys, we calculated $a_{s,t} = L_{s,t} * 1.2/A_{s,t}$, where $L_{s,t}$ was the length of transect flown in cell s at time t, and the 1.2 represents a fixed width of 0.6 km on either side of the aircraft (which is motivated by the distribution of observed polar bear distances; see below). We set $\theta_{s,t} = p_{us}$ for U.S. surveys and $\theta_{s,t} = p_{rus}$ for Russian surveys, which are detection probability of bears for U.S. and Russian surveys, respectively.

For U.S. surveys, information about detection probability, p_{us} was obtained from experimental flyovers of polar bears. In particular, we assumed that the number of detected bears in experimental flyovers (D) was binomially distributed with an index equal to the number of first flyovers (n=12) and success probability p_{us}:

$$D \sim \text{Binomial}(n, p_{us}).$$

For Russian surveys, we used distance sampling ideas to model detection probability. In particular, we write

$$p_{rus} = g(0) \int_0^w \frac{h(x)}{wh(0)} \, dx,$$

where $h(x)$ is a probability density function describing the distribution of observed distances (using either in situ observations of distance made using an inclinometer or post hoc examination of photographs) from the transect line, and $g(0)$ gives the probability of detection on the transect line. We fitted a number of detection functions to distance data, and determined that the half normal distribution adequately characterizes the decline in detections as a function of distance (Supplementary Appendix A). Note that our comparison of detection functions included an option for a uniform-half normal mixture, where the uniform distribution applied to distances of 350 m from the aircraft and the normal distribution applied out to the truncation distance of $w = 600$ m. Since photographs were only obtained up to a fixed distance of the aircraft depending on altitude (median = 350 m), one might expect this mixture detection function to better apply to our data (photographs + human observers), but the half-normal was favored by AIC (ΔAIC = 1.6). We thus used it as the functional form of h, treating $\log(\sigma)$ (the log of the half normal standard deviation) as an unknown parameter.

The primary challenge with this approach is that data were not gathered in a manner amenable to estimating $g(0)$, the proportion of animals that are seen on the transect line (i.e., underneath the aircraft). Although $g(0)$ from other polar bear surveys are available (and most are close to 1.0; Supplementary Table 1), most of these studies used helicopters traveling at substantially slower speeds and lower altitudes than the fixed wing aircraft used in Russian surveys. Many of these surveys were also conducted in fall over snow-free land where bears are more visible (Supplementary Appendix A). For these reasons, in situ detection probability of human observers was potentially much lower than reported in the literature. On the other hand, we also incorporated detections of bears from post hoc examinations of photographs into our distance sampling analysis which markedly increased sample size and raised $g(0)$ relative to it's value had we just used in situ observations. Given these confounding factors, it is difficult to make an objective determination of a likely value for $g(0)$. We

thus tried a range of values including $g(0) = 0.6$, 0.8, and 1.0.

If we limited analysis to counts made by thermal cameras while "on effort" for U.S. surveys we would be limited to $n = 3$ sightings. However, we also had access to an additional $n = 5$ detections of bear groups that were detected visually while on transects (often beyond the range of our thermal scanners). However, we did not collect distance measurements in U.S. surveys, so there was no way to calculate effective strip width [28]. As such, we did not know the effective area surveyed nor have a good idea of detection probability for this class of observations. Nevertheless, these data are clearly informative about the locations and habitats that bears were detected and we wished to use them to improve estimation of density-habitat relationships. To incorporate these data, we modeled these auxiliary bear group counts ($U_{s,t}$) as

$$U_{s,t} \sim \text{Poisson}(\xi_{us} N \pi_{s,t} p_{s,t}),$$

where ξ_{us} is an estimated parameter representing the relative detectability of auxiliary counts relative to thermal detections in U.S. surveys. We assumed this ratio remained constant over the course of the survey.

Thus far, we have described models for the abundance of polar bear groups, as opposed to total abundance. This is because we viewed groups as the primary sampling unit. We estimated absolute abundance, N^*, as $N^* = \mu_g N$, where μ_g is a parameter representing absolute group size. We modeled observed group size (g_i) for each of $i = 1, 2, \cdots, 49$ groups as a realization from a zero-truncated Poisson process; that is,

$$(g_i - 1) \sim \text{Poisson}(\mu_g - 1).$$

Models and model fitting

We fitted three different models to count and detection data, corresponding to three assumed values for $g(0)$ in Russian surveys: 0.6, 0.8, or 1.0. The model for polar bear tracks was relatively simple and used simple polynomial regression such that $\mathbf{x}'_{s,t}$ included an intercept, linear and quadratic effects of distance from land, linear and quadratic effects of sea ice concentration, and a linear effect of the resource selection function value (RSF) for a given cell.

Models for latent polar bear abundance were considerably more complex, as we wanted the data to "speak for themselves" by allowing smooth effects of covariates as commonly employed in modern density surface modeling of species distributions [29]. To that end, we adopted a penalized spline formulation for smooth effects [30]. First, we used the `mgcv` package [31] in the R programming environment [32] to construct cubic smoothing bases and penalty matrices. We set the maximum basis dimension for each covariate to six to reduce dimensionality; each smooth effect thus included six parameters. Following the approach implemented in the 'jagam' function in the `mgcv` package [33]. we implemented penalties by imposing prior distributions on the regression coefficients, $\boldsymbol{\alpha}$. In particular,

$$\boldsymbol{\alpha} \sim \text{Multivariate normal}(\mathbf{0}, \boldsymbol{\Sigma}),$$

where $\boldsymbol{\Sigma}$ is a block diagonal matrix with block entries given by $\lambda_i \mathbf{S}_i$. Here, $\mathbf{S}_i$ gives a penalty matrix output from `mgcv` and λ_i is a parameter that controls the degree of smoothing. As in the 'jagam' function, we assigned Gamma prior distributions to the λ_i parameters:

$$\lambda_i \sim \text{Gamma}(0.05, 0.005).$$

In bear count models, we included latent polar bear track intensity ($Z_{s,t}$) as an additional linear fixed effect. Inference about N thus properly accounts for uncertainty in the relative frequency of bear tracks. In order to prevent our model from predicting polar bears or tracks in grid cells without (or with very little) sea ice, we included the covariate `Water99` in all models fitted, and set the corresponding regression coefficient equal to -50.0. For reference, all but two sightings of polar bears occurred in grid cells with sea ice concentrations $> 80\%$ (although one bear was observed in a cell with $\approx 3\%$ ice).

In order to propagate uncertainty from track, count, detection probability, and group size models into final abundance estimates, we based statistical inference for polar bear abundance on the joint marginal likelihood

$$L = \int_{\boldsymbol{\alpha}} \int_{\boldsymbol{\lambda}} L_t L_c L_u L_p L_g P_\alpha P_\lambda \; d\boldsymbol{\alpha} \; d\boldsymbol{\lambda},$$

where L_t represents the likelihoods for U.S. and Russian polar bear tracks, L_c denotes Poisson likelihoods for instrument-based and distance-sampling counts (for U.S. and Russian surveys, respectively), L_u denotes a Poisson likelihood for U.S. human observer counts, L_p denotes binomial and half normal likelihoods for U.S. flyovers and Russian distance data, L_g is the zero-truncated Poisson likelihood for group size, and P_α and P_λ give prior distributions for spline parameters $\boldsymbol{\alpha}$ and $\boldsymbol{\lambda}$, respectively. We treated spline parameters as random effects, and integrated them out of the likelihood using the Laplace approximation capability in Template Model Builder [34] (see *Software* below).

Log and logit link functions were used on bounded parameters to allow unbounded optimization. Point estimates and Hessian-based standard errors were used to construct 95% log-based confidence intervals [28, 35] for total abundance.

Goodness-of-fit

To examine the fit of track and bear count submodels, we examined distributions of randomized quantile residuals (RQRs) [36]. Such residuals are especially useful for discrete random variables, where it can be difficult to assess lack-of-fit visually because of "clumping" at fixed values. For an arbitrary datum y_i, randomized quantile residuals were simulated as

$$R_i = F(y_i|\hat{\mu}_i) + u_i f(y_i|\hat{\mu}_i),$$

where $F()$ gives a cumulative distribution function (CDF) with mean $\hat{\mu}_i$, u_i is a uniform random deviate, and $f()$ is a probability mass function (PMF). For example, for a Poisson count model, y_i represents an observed count, $\hat{\mu}_i$ represents a predicted count, and $F()$ and $f()$ give the Poisson CDF and PMF, respectively. If a model fits the data well, RQRs computed in this way should be uniformly distributed on (0,1). To assess uniformity, we calculated chi-squared test statistics with 10 equally sized bins for each count or track submodel.

Software

We programmed our joint likelihood in the templated C++ code structure required to conduct inference with the `TMB` package [34] for the R computing environment [25].

Results

In the U.S., we flew 15720 km and photographed 5830 km^2 of sea ice habitat during "on effort" portions of U.S. surveys (e.g., excluding thick fog or banking turns). The thermal swath of the Russian infrared scanner covered ≈ 5414km^2 in flights totalling 11604 km. We detected three groups of polar bears in U.S. waters using our thermal detection algorithm (Fig 1), and visually detected another five groups (Fig 1). Russian survey flights detected five groups of bears using thermal sensors, and another 44 groups using a combination of human visual detections and manual searches of photographs. Mean group size of polar bears in U.S. surveys was 1.25 (SE 0.25) and 1.39 (SE 0.10) in Russian surveys. For mothers with dependent young, the mean number of observed dependents was 1.4 (SE 0.13).

In U.S. thermal detection trials, our thermal detection algorithm correctly identified 8 of 12 bear groups for a detection probability of $\hat{p} = 0.67$. The Malachite-M thermal sensor used in Russian surveys did not detect any bears when temperatures were $< -5°$ C; even in warmer temperatures it only detected 1 of 9 bear groups that were seen by human observers and deemed to be within the thermal detection swath.

Our joint models for polar bear abundance and tracks produced abundance estimates ranging from $\hat{N} = 3,555$ (95% CI: 2,382-5,305) to $\hat{N} = 5,623$ (95% CI: 3,761-8,408) depending on the proportion of bears assumed to be missed on the transect line during Russian surveys (Table 1). Our models fit polar bear counts well, but considerable lack-of-fit was discernible in models for bear tracks (Fig 2). Such lack-of-fit was likely an artifact of trying to join different types of observations (on the U.S. side: systematic sampling of photographs; on the Russian side: subjective determinations of the number of distinct tracks made by human observers). Although the spatial distribution of polar bear tracks looked similar to estimated bear densities (Fig. 3), it did not help explain bear abundance better than other physiographic variables. In particular, after accounting for effects of other covariates (Fig 4), confidence intervals for track effect estimates considerably overlapped zero (e.g., $\alpha_{\text{tracks}} = -0.55$, SE 0.66). Absolute correlation between estimated track density and other explanatory variables was relatively low (< 0.51).

Distribution maps suggested higher abundance and track densities within several

Fig 2. Goodness-of-fit diagnostics. Randomized quantile residuals (RQRs) for assessing goodness-of-fit for the most highly parameterized model fit to polar bear encounter data. RQRs should be uniformly distributed on (0,1) for a well-fitting model. Also presented are χ^2 test p-values to assess uniformity.

Fig 3. Spatio-temporal maps of sea ice and predicted polar bear distribution. Remotely sensed sea ice concentration values (top row), estimated polar bear track index (middle row), and predictions of polar bear abundance at the beginning, middle, and end of 2016 aerial surveys of the eastern Chukchi Sea ($g(0) = 0.8$ scenario). The polar bear track index is an estimate of the proportion of photographs that would contain polar bear tracks had photographs been taken in all grid cells and on all days of the survey. Predicted abundance ('N') is calculated as $\hat{N}_{s,t} = \hat{N}\hat{\pi}_{s,t}\mu_g$.

Fig 4. Covariate effects. Estimated smooth effects of covariates on polar bear abundance (black line), together with 95% confidence intervals (grey shading). Note that distance from land, easting, and northing effects were standardized to have a mean of 1.0 prior to analysis. Polar bear tracks were modeled as a simple linear effect on abundance so do not appear here.

hundred kilometers from land when sea ice is present (Fig 3), and relatively low densities far out on the pack ice. Our models predicted polar bear abundance to be substantially (close to ten times) higher in Russian than U.S. waters (Table 1). Point estimates of abundance in the Regehr et al. (2018) intensive capture-recapture study area were 99-114 depending upon $g(0)$ assumption (Table 1). By contrast, an integrated population model produced a posterior mean of 78 (95% credible interval 36-138) bears in this region [13], which was well within the confidence intervals of our predictions.

Translation of polar bear abundance to density is somewhat complicated by the fact that sea ice changed considerably throughout the survey. Calculating the area of sea ice habitat as $\text{ice}_t = \sum_s \text{ice}_{st}$ for time t and spatial grid cell s leads to values ranging from 824,000 km^2 at the beginning of the study to 610,000 km^2 at the end of the study. Conservative point estimates of densities (i.e., for $g(0) = 1.0$) therefore ranged from 0.004 bears/km^2 of sea ice habitat at the beginning of the study to 0.006 bears/km^2 at the end of the study. Making this same calculation with respect to international boundaries results in conservative point estimates of 0.011 - 0.020 bears/km^2 of sea ice in Russian waters and 0.001-0.002 bears/km^2 in U.S. waters during spring of 2016.

Table 1. Estimates of polar bear abundance for different regions and assumptions about $g(0)$ in Russian survey flights, along with 95% log-based confidence intervals. Regions include the full study grid ('Chukchi'), mean (i.e., time-averaged) abundance for those cells of the survey grid with centroids in U.S. waters ('U.S.'), mean abundance in Russian waters ('Russia'), mean abundance in the portions of our study area that overlapped the polar bear specialist group boundary ('PBSG'), and an estimate for the area used for capture and release of bears in a mark-recapture study of polar bears in the Chukchi Sea ('Regehr' [13]). For a map of the study area and related regions, see Fig. 1).

Region	$g(0)$	$\hat{N}^*$	95% CI
Chukchi	1.0	3555	(2382-5305)
Chukchi	0.8	4339	(2906-6480)
Chukchi	0.6	5623	(3761-8408)
U.S.	1.0	345	(146-813)
U.S.	0.8	370	(158-868)
U.S.	0.6	404	(173-944)
Russia	1.0	3210	(2136-4825)
Russia	0.8	3969	(2638-5973)
Russia	0.6	5219	(3464-7864)
PBSG	1.0	3236	(2196-4768)
PBSG	0.8	3952	(2680-5827)
PBSG	0.6	5123	(3471-7564)
Regehr	1.0	99	(40-245)
Regehr	0.8	106	(43-260)
Regehr	0.6	114	(46-281)

Discussion

Our study represents the first comprehensive attempt at using aerial surveys to estimate polar bear abundance in the Chukchi Sea region. Polar bears are difficult to enumerate because they occur at low densities over large geographical areas with few human settlements from which to execute surveys. Long-term (i.e., approximately 35 years, which corresponds to three polar bear generations [37]) population trends are only available for 7 of 19 subpopulations worldwide [38]. Previous estimates of polar bear abundance have primarily been based on intensive physical or genetic capture-recapture surveys conducted over multiple years [39–42], or on autumn distance sampling surveys using helicopters in regions where polar bears occur at high densities on land [43–45]. Instead, our surveys were conducted with fixed wing aircraft at faster speeds, higher altitudes, and with greater fuel capacity. Conducting instrument-based surveys in the spring provided a permanent record of fine-scale sea-ice habitat and data on distribution and abundance of key prey species such as ringed and bearded seals. Such

surveys (ideally using automated infared detection) are therefore an attractive option, given the potential for increased efficiency, longer ranges, reduced disturbance to bears, increased safety of survey personnel, and comprehensive data collection. Note, however, that unlike physical capture studies, such "snapshot" surveys do not provide inference about health or demographic processes such as survival, so we do not recommend them as a replacement for intensive capture-recapture experiments.

Our intention was to base estimation on infrared detections made throughout the surveyed area. However, the thermal sensors employed in U.S. and Russian surveys performed differently. The algorithm applied to thermal imagery collected during U.S. surveys detected 67% of polar bear groups in experimental trials, whereas the Malachite-M sensor used in the Russian survey never detected bears below $-5°$ C; even above $-5°$ C, very few bears were encountered compared to other modes of detection (e.g., visual detections by crew, post hoc manual examination of photographs). As such, we used distance sampling methods to model detection of bears in Russian surveys. This approach requires assumptions about the proportion of bears detected on the transect line ($g(0)$), for which empirical estimates were unavailable.

Our model-based estimates of abundance varied considerably based on the $g(0)$ value assumed for Russian surveys. For example, under the optimistic scenario that 100% of bears were detected on the transect line ($g(0) = 1.0$), total estimated abundance in our study area was 3,555 (95% CI: 2,382-5,305) for an area bounded by Chaunskaya Bay, Russia, to the west and Point Barrow, Alaska to the east (Fig 1). We suggest this number represents a minimum plausible estimate given that lower values of $g(0)$ will produce higher abundance estimates. For example, use of $g(0) = 0.6$ produced an estimate of 5,623 (95% CI: 3,761-8,408). Although these values vary considerably, they are of similar magnitude to that produced by a recent integrated population model fitted to data from a 2018-2016 live-capture study near Kotzebue, Alaska, USA, and extrapolated to other portions of the CS subpopulation boundary ($\hat{N} = 2,937$; 95% CI 1522-5944) [13]. The integrated population model was presumably most reliable in the area where physical marking and recapture events took place. Aerial survey estimates in this smaller region (Fig. 1, Table 1) were similar to (and slightly larger than) those estimated from the integrated population model (78 bears [13]). Considering changes in sea-ice area during the survey, and conservatively assuming $g(0) = 1.0$, estimates of

absolute density ranged from 0.004–0.006 bears/km^2 in our study area (U.S. + Russia). These values are similar to a mean estimate of 0.0036 (95% credible interval = 0.0019–0.0073) for the period 2008–2016 within the CS subpopulation boundary, based on an integrated population model with density extrapolation [13]; and to a mean estimate of 0.0024 (range of point estimates 0.0006–0.0093) for 14 polar bear subpopulations with available data, as reviewed by Hamilton and Derocher [46].

Given the impact of $g(0)$ on abundance estimation, it is important to review available data that might inform a likely range of values. Multiple-observer polar bear surveys reported in the literature have typically resulted in high $g(0)$ values (e.g., 0.85 - 0.90), although these estimates are mostly based on helicopters flying at lower speeds, lower altitudes, and over land (Supplementary Appendix A). Detection rates from these studies were thus likely to be higher than those for in situ observations made during Russian survey flights because helicopter observers are closer to animals and have increased visual contrast compared to observations made fixed wing aircraft flying over sea ice. However, in addition to human detections made from the air (which would be expected to have lower $g(0)$ values than reported in the literature), we also detected a relatively large number of bears through post hoc visual inspection of photographs and incorporated these into our distance sampling analysis. Conditions being equal, this process should increase $g(0)$ relative to multiple observer surveys. Owing to the differences in survey protocols used here versus reported in the literature, we are currently unable to provide advice on which value of $g(0)$ is most appropriate, and suggest it be a topic for future research.

Several other assumptions of our modeling approach deserve further discussion. For instance, we assumed that the total number of bears within our study area was constant as surveys were being conducted. Our modeling framework allowed bears to redistribute with changing conditions (e.g., as sea ice melted), but not to move in or out of the study area. Simulation studies have shown this assumption helps stabilize abundance estimates [26], and was crucial in this application given the low degrees of freedom. As such, our estimates are best interpreted as the average number of bears in the study area while sampling was being conducted.

We also assumed that bears selected for experimental flyovers were representative of the population with respect to infrared detectability. We were initially concerned that

estimates of detection probability were biased high because detection trials were performed for a small number of animals visible to human observers (e.g., trial bears were usually moving and more often on flat ice than rubble). However, subsequent testing of infrared cameras (using captive bears and during additional flights in 2019) suggest that bears are similarly detectable in different behavioral states except after recently emerging from water (E. Moreland, unpublished data). Potential methods to improve automated detection include additional sensors (e.g., UV spectrum) and approaches to find and classify bears in imagery (e.g., artificial intelligence).

Our analysis incorporated covariates such as RSFs and polar bear track indices as predictors of polar bear abundance within a generalized additive modeling framework. Development of track indices required their own modeling exercise; however, we propagated uncertainty in the estimated index into the analysis for bear abundance through a joint likelihood modeling framework. To our knowledge, this is the first attempt to use tracks to predict polar bear abundance. Use of RSFs and tracks as predictors of abundance requires several caveats. For instance, RSFs were based on a relatively small sample of non-denning adult females fitted with radiocollars [8], which may not represent habitat use of the larger subpopulation. Similarly, polar bear tracks indicate presence of bears at some point in time, but the longevity, visibility, and location of tracks was likely a function of many factors including sea-ice dynamics, surface characteristics (e.g., hard ice vs. deep snow), and weather. Although further investigation with more consistent track sampling protocols would be worthwhile, the lack of support for models that incorporated track density as predictors of polar bear abundance suggests that there utility for population monitoring (i.e., as a relative abundance index) may be limited.

Our experiences with instrument-based aerial surveys for polar bears led to several suggestions for future improvement. First, infrared sensors do not all reliably detect polar bears and the instrument used in Russian surveys should be replaced with a more reliable model. Second, we suggest that for a given sensor or sensor array (e.g., IR and UV) a designed study should be performed to obtain a reliable estimate of detection probability for use in abundance estimation. In U.S. surveys, we conducted experimental flyovers of bears which yielded a serviceable estimate. However, in Russian surveys no independent estimate was available. Third, if future surveys use distance

sampling methods, mark-recapture distance sampling with two observers [47–49] may provide an estimate of $g(0)$. However, such methods require statistical independence of observations, at least at a single distance value (if point independence is assumed [48, 50]), which may be violated if both observers key into unique features of animals to aid detection (e.g., distinctiveness, movement). As such, an independent estimate of $g(0)$ derived from a different detection approach is highly desirable. For instance, if infrared detections are automated, the proportion of animals also detected by human observers provides an independent estimate of detection probability. Unfortunately, in our surveys, all forms of detection in Russian surveys were dependent (for instance, photographs were often triggered when humans detected animals) and such "double sampling" estimates of detection probability would have been inappropriate. If double sampling is used in future surveys to estimate detection probability, some level of autonomy is needed between infrared detections, photographs, and human observers. Finally, we did not originally intend to collect data on polar bear tracks, which led to different protocols in U.S. and Russian surveys. Ideally, the same protocol (e.g., systematic sampling of automated photographs as used on the U.S. side) would be used to collect data on polar bear tracks. Although we used a spatial point process model to join these two data sources, the fit of this model was poor, particularly for Russian track data.

Conclusion

Despite the methodological and technical challenges in this initial application, we are cautiously optimistic about the use of instrument-based aerial surveys for polar bears. Our approach has the potential to estimate distribution and abundance with minimal disturbance to bears and less risk to human safety relative to existing methods. The increased range of fixed-wing aircraft used in our surveys could increase access to very remote subpopulations where sample sizes from helicopter-based studies have been prohibitively small (e.g., Kane Basin [51]). Furthermore, instrument-based surveys provide information on the distribution and abundance of ice-dependent seals, the primary prey of polar bears [52]. Recent studies have shown that relationships between sea-ice conditions and polar bear demography are variable in time and space [42, 53, 54],

emphasizing that additional data on trophic relationships and ecosystem function are important to understanding the effects of climate change on polar bears. Refinement of future survey protocols, implementation of detection algorithms trained specifically on polar bears, and improved estimates of detection probability will increase the reliability and precision of abundance and distribution estimates derived from instrument-based aerial surveys.

Acknowledgments

We thank all researchers and technicians who participated in ChESS surveys, processed imagery, and organized data. In the U.S, this included G. Brady, S. Brown, M. Cameron, C. Christman, S. Dahle, S. Hardy, B. Hou, C. Johnson, E. Richmond, and A. Willoughby. In Russia, this included D. Glazov (Severtsov Institute) and Dr. D. Litovka (Government of Chukotka). We thank N. Chernook and V. Asyutenko (ANO Ecofactor) for image processing and Dr. N. Platonov (Severtsov Institute) for survey on-the-ground support. Dr. V. Burkanov (NPWC) provided overall supervision of the Russian survey planning and implementation. Financial and logistical support of the survey in Russia was provided by Marine Mammal Council and WWF Russia. Data analysis was carried out with support from the North Pacific Research Board and The Prince William Sound Oil Spill Recovery Institute subaward NA17NMF4720289, project 1813. Views and conclusions in this article represent the views of the authors but do not necessarily represent findings or policy of the U.S. National Oceanic and Atmospheric Administration or U.S. Fish & Wildlife Service. Any use of trade, firm, or product names is for descriptive purposes only and does not imply endorsement by the U.S. Government.

References

1. Durner GM, Laidre KL, York GS. Polar Bears: Proceedings of the 18th Working Meeting of the IUCN/SSC Polar Bear Specialist Group, 7–11 June 2016, Anchorage, Alaska. Gland, Switzerland and Cambridge, UK: IUCN. 2018;.

2. USFWS. Endangered and Threatened Wildlife and Plants; Determination of Threatened Status for the Polar Bear (Ursus maritimus) throughout its Range. United States Fish And Wildlife Service. Fed Regist. 2008; p. 28212–28303.
3. Atwood TC, Marcot BG, Douglas DC, Amstrup SC, Rode KD, Durner GM, et al. Forecasting the relative influence of environmental and anthropogenic stressors on polar bears. Ecosphere. 2016;7(6):e01370.
4. Stern HL, Laidre KL. Sea-ice indicators of polar bear habitat. Cryosphere. 2016;10(5).
5. Voorhees H, Sparks R, Huntington HP, Rode KD. Traditional knowledge about polar bears (Ursus maritimus) in Northwestern Alaska. Arctic. 2014; p. 523–536.
6. Regehr EV, Wilson RR, Rode KD, Runge MC, Stern HL. Harvesting wildlife affected by climate change: a modelling and management approach for polar bears. J Appl Ecol. 2017;54(5):1534–1543.
7. Taylor M, Lee J. Distribution and abundance of Canadian polar bear populations: a management perspective. Arctic. 1995; p. 147–154.
8. Wilson RR, Regehr EV, Rode KD, St Martin M. Invariant polar bear habitat selection during a period of sea ice loss. Proceedings of the Royal Society B: Biological Sciences. 2016;283(1836):20160380.
9. Belikov SE. Number, distribution, and migrations of polar bear in the Soviet Arctic. Krupnye Khishniki (Big Predators) Moskva, CNIL Glavokhoty RSFSR. 1992; p. 74–84.
10. Vaisfeld MA, Chestin IE. Bears: brown bears, polar bears, Asian black bear. Distribution, ecology, use, and protection. Game animals of Russia and adjacent countries and their environment. Moscow: Nauka; 1993.
11. Rode KD, Regehr EV, Douglas DC, Durner G, Derocher AE, Thiemann GW, et al. Variation in the response of an Arctic top predator experiencing habitat loss: feeding and reproductive ecology of two polar bear populations. Global Change Biol. 2014;20(1):76–88.

12. Rode KD, Wilson RR, Douglas DC, Muhlenbruch V, Atwood TC, Regehr EV, et al. Spring fasting behavior in a marine apex predator provides an index of ecosystem productivity. Global Change Biol. 2018;24(1):410–423.

13. Regehr EV, Hostetter NJ, Wilson RR, Rode KD, Martin MS, Converse SJ. Integrated population modeling provides the first empirical estimates of vital rates and abundance for polar bears in the Chukchi Sea. Sci Rep. 2018;8(1):16780.

14. Regehr EV, Polasek L, Von Duyke A, Wilder JM, Wilson RR. Harvest Risk Assessment for Polar Bears in the Chukchi Sea: Report to the Commissioners of the U.S.-Russia Polar Bear Agreement; 2018.

15. Chernook VI, Kuznetsov NB, Yakovenko MY. Multispectral aerial survey of haulouts of ice seals. Murmansk, Russia: PINRO Publications; 1999.

16. Speckman SG, Chernook VI, Burn DM, Udevitz MS, Kochnev AA, Vasilev A, et al. Results and evaluation of a survey to estimate Pacific walrus population size, 2006. Mar Mammal Sci. 2011;27:514–553.

17. Conn PB, Ver Hoef JM, McClintock BT, Moreland EE, London JM, Cameron MF, et al. Estimating multi-species abundance using automated detection systems: ice-associated seals in the eastern Bering Sea. Methods Ecol Evol. 2014;5:1280–1293.

18. Amstrup SC, York G, McDonald TL, Nielson R, Simac K. Detecting denning polar bears with forward-looking infrared (FLIR) imagery. Bioscience. 2004;54:337–344.

19. Diggle PJ, Menezes R, Su Tl. Geostatistical inference under preferential sampling. Journal of the Royal Statistical Society: Series C (Applied Statistics). 2010;59(2):191–232. doi:10.1111/j.1467-9876.2009.00701.x.

20. Conn PB, Thorson JT, Johnson DS. Confronting preferential sampling when analysing population distributions: diagnosis and model-based triage. Methods Ecol Evol. 2017;8(11):1535–1546.

21. Diggle P, Lophaven S. Geostatistical Design. Scand J Stat. 2006;33:53–64.

22. Preciado J, Rubinsky B, Otten D, Nelson B, Martin M, Greif R. Radiative Properties of Polar Bear Hair. ASME International Mechanical Engineering Congress and Exposition, Proceedings. 2002;53:1–2.

23. Havens JK, Sharp EJ. Thermal Imaging Techniques to Survey and Monitor Animals in the Wild. London, U.K.: Academic Press; 2016.

24. Hiemstra PH, Pebesma EJ, Twenhofel CJW, Heuvelink GBM. Real-time automatic interpolation of ambient gamma dose rates from the Dutch radioactivity monitoring network. Computers & Geosciences. 2009;35:1711–1721.

25. R Development Core Team. R: A Language and Environment for Statistical Computing; 2016. Available from: `http://www.R-project.org`.

26. Conn PB, Johnson DS, Ver Hoef JM, Hooten MB, London JM, Boveng PL. Using spatio-temporal statistical models to estimate animal abundance and infer ecological dynamics from survey counts. Ecological Monographs. 2015;85:235–252.

27. Boyd C, Barlow J, Becker EA, Forney KA, Gerrodette T, Moore JE, et al. Estimation of population size and trends for highly mobile species with dynamic spatial distributions. Divers Distrib. 2018;24(1):1–12. doi:10.1111/ddi.12663.

28. Buckland ST, Anderson DR, Burnham KP, Laake JL, Borchers DL, Thomas L. Introduction to Distance Sampling: Estimating the abundance of biological populations. Oxford, U.K.: Oxford University Press; 2001.

29. Miller DL, Burt ML, Rexstad EA, Thomas L. Spatial models for distance sampling data: recent developments and future directions. Methods Ecol Evol. 2013;4:1001–1010.

30. Skaug HJ. pSplines GitHub repository; 2019. `https://github.com/skaug/tmb-case-studies/tree/master/pSplines`.

31. Wood SN. Generalized additive models, 2nd edition. Boca Raton, Florida: Chapman & Hall/CRC; 2017.

32. R Development Core Team. R: A Language and Environment for Statistical Computing; 2019. Available from: `http://www.R-project.org`.

33. Wood SN. Fast stable restricted maximum likelihood and marginal likelihood estimation of semiparametric generalized linear models. Journal of the Royal Statistical Society (B). 2011;73:3–36.

34. Kristensen K, Nielsen A, Berg CW, Skaug H, Bell BM. TMB: Automatic differentiation and Laplace approximation. Journal of Statistical Software. 2016; p. doi: 10.18637/jss.v070.i05.

35. Burnham KP, Anderson DR, White GC, Brownie C, Pollock KH. Design and analysis methods for fish survival experiments based on release-recapture. American Fisheries Society Monograph. 1987;5:1–437.

36. Dunn PK, Smyth GK. Randomized quantile residuals. Journal of Computational and Graphical Statistics. 1996;5(3):236–244.

37. Regehr EV, Laidre KL, Akçakaya HR, Amstrup SC, Atwood TC, Lunn NJ, et al. Conservation status of polar bears (Ursus maritimus) in relation to projected sea-ice declines. Biol Lett. 2016;12(12):20160556.

38. Polar Bear Specialist Group. Summary of polar bear population status per 2019; 2019. `http://pbsg.npolar.no/en/status/status-table.html`.

39. Regehr EV, Lunn NJ, Amstrup SC, Stirling I. Effects of earlier sea ice breakup on survival and population size of polar bears in western Hudson Bay. J Wildl Manage. 2007;71(8):2673–2683.

40. Taylor MK, Laake J, McLoughlin PD, Cluff HD, Messier F. Mark-recapture and stochastic population models for polar bears of the High Arctic. Arctic. 2008; p. 143–152.

41. Peacock E, Taylor MK, Laake J, Stirling I. Population ecology of polar bears in Davis Strait, Canada and Greenland. The Journal of Wildlife Management. 2013;77(3):463–476.

42. Bromaghin JF, McDonald TL, Stirling I, Derocher AE, Richardson ES, Regehr EV, et al. Polar bear population dynamics in the southern Beaufort Sea during a period of sea ice decline. Ecol Appl. 2015;25(3):634–651.

43. Aars J, Marques T, Buckland S, Andersen M, Belikov S, Boltunov A, et al. Estimating the Barents Sea polar bear subpopulation size. Mar Mammal Sci. 2009;25(1):35–52.

44. Stapleton S, Atkinson S, Hedman D, Garshelis D. Revisiting Western Hudson Bay: Using aerial surveys to update polar bear abundance in a sentinel population. Biol Conserv. 2014;170:38–47.

45. Obbard ME, Stapleton S, Middel KR, Thibault I, Brodeur V, Jutras C. Estimating the abundance of the Southern Hudson Bay polar bear subpopulation with aerial surveys. Polar Biol. 2015; p. 1–13.

46. Hamilton SG, Derocher AE. Assessment of global polar bear abundance and vulnerability. Anim Conserv. 2019;22:83–95.

47. Borchers D, Zucchini W, Fewster RM. Mark-recapture models for line transect surveys. Biometrics. 1998;54:1207–1220.

48. Buckland ST, Laake JL, Borchers DL. Double-observer line transect methods: Levels of independence. Biometrics. 2010;66:169–177.

49. Burt ML, Borchers DL, Jenkins KJ, Marques TA. Using mark–recapture distance sampling methods on line transect surveys. Methods Ecol Evol. 2014;5(11):1180–1191.

50. Borchers DL, Laake JL, Southwell C, Paxton CGM. Accomodating unmodeled heterogeneity in double-observer distance sampling surveys. Biometrics. 2006;62:372–378.

51. SWG. Re-assessment of the Baffin Bay and Kane Basin polar bear subpopulations: final report to the Canada-Greenland joint commission on polar bear; 2016.

52. Amstrup SC. Polar Bear (*Ursus maritimus*). In: Feldhamer GA, Thompson BC, Chapman JA, editors. Mammals of North America: Biology, Management, and Conservation. Baltimore, Maryland: John Hopkins University Press; 2003. p. 587–610.

53. Lunn NJ, Servanty S, Regehr EV, Converse SJ, Richardson E, Stirling I. Demography of an apex predator at the edge of its range: impacts of changing sea ice on polar bears in Hudson Bay. Ecol Appl. 2016;26(5):1302–1320.

54. Laidre KL, Atkinson S, Regehr EV, Stern HL, Born EW, Wiig Ø, et al. Interrelated ecological impacts of climate change on an apex predator. Ecol Appl. 2020; p. e02071.

 Click here to access/download;Figure;Effort_big.tiff

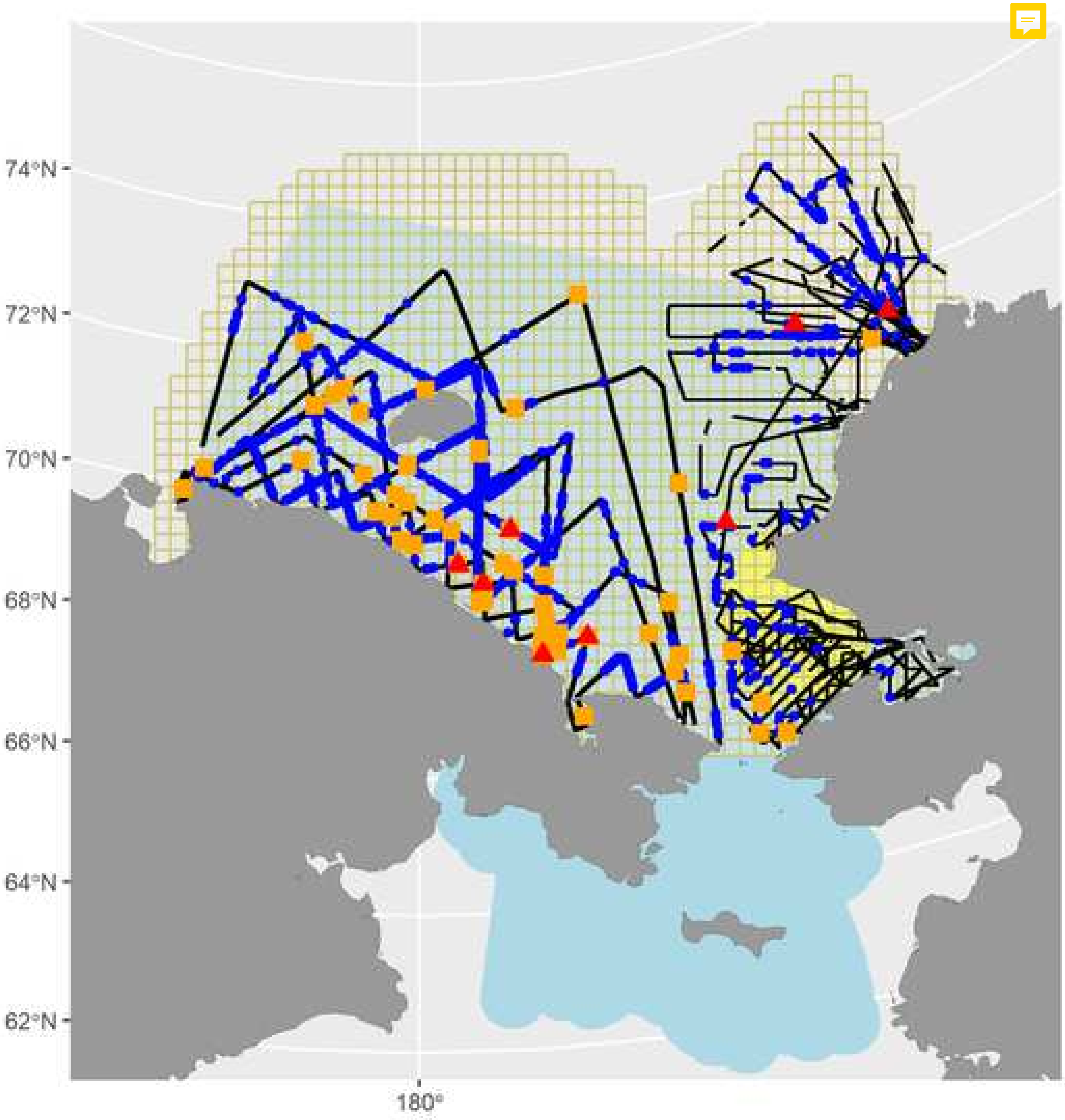

Figure
Click here to access/download;Figure;GOF_bears_tracks.tiff

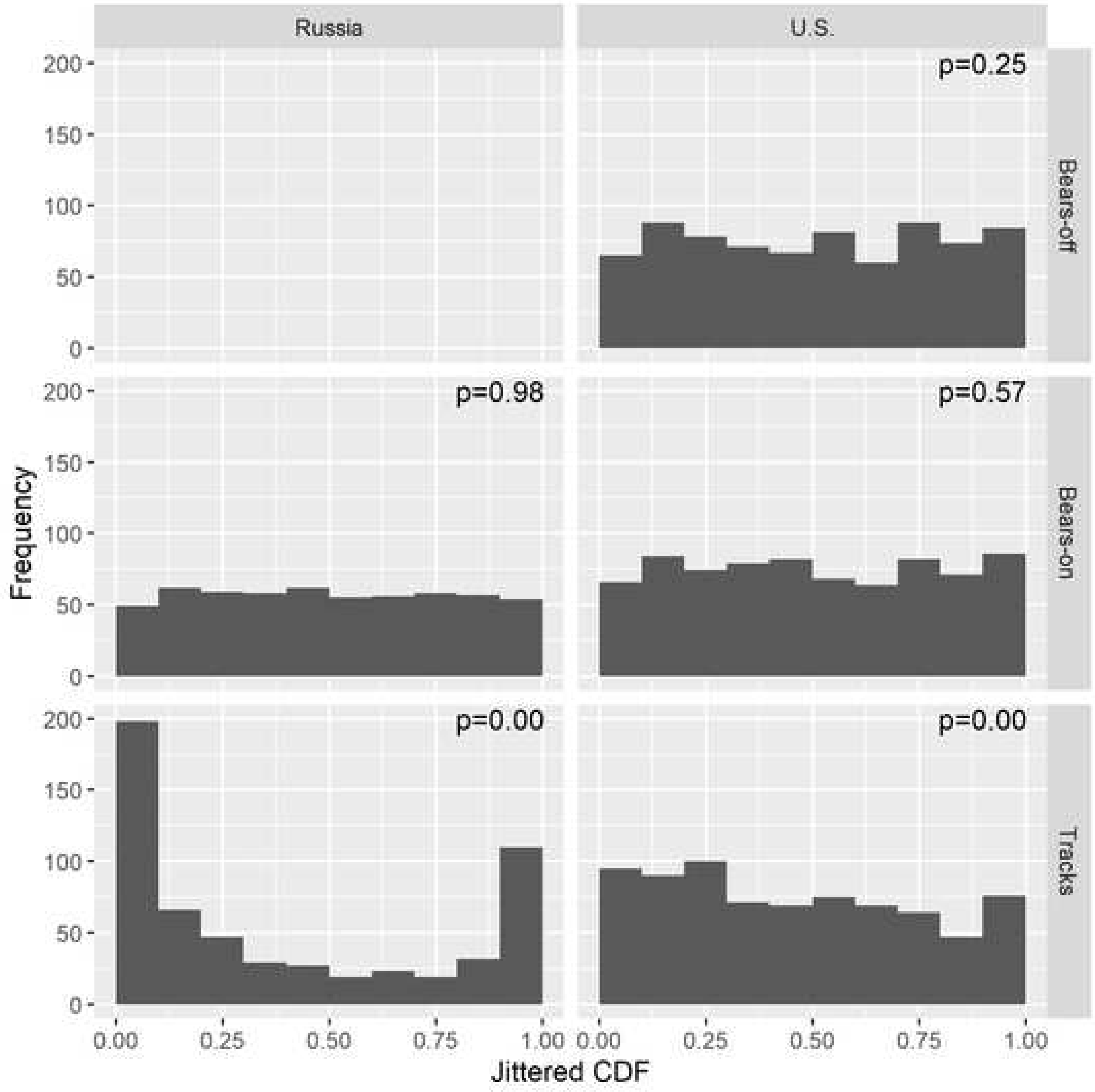

Click here to access/download;Figure;IceTracksAbund_spatial.tiff

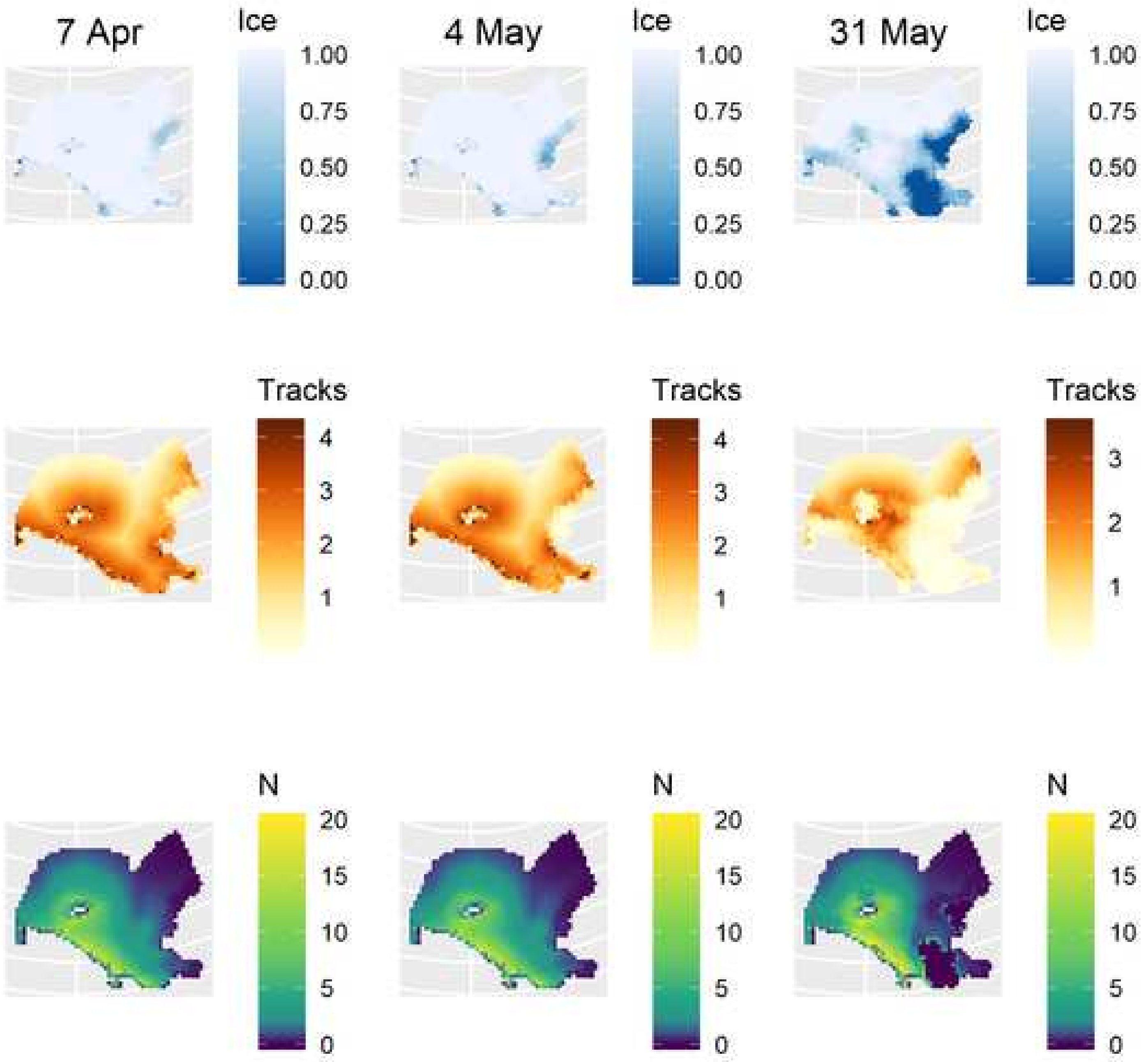

 Click here to access/download;Figure;pb_spatial_smooths.pdf.tiff

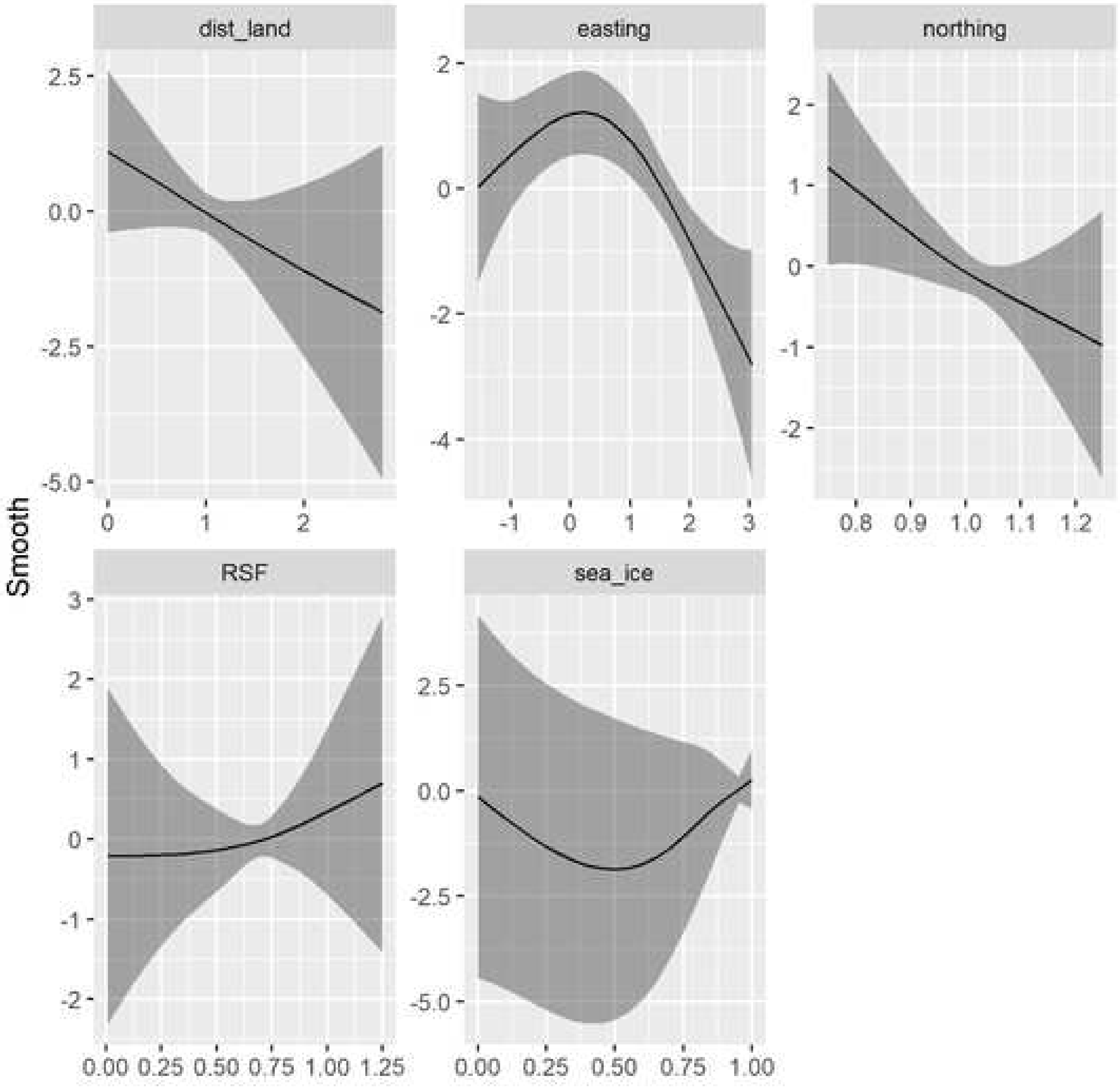

Supplement: Attachment — Submitted filename: PONE-D-20-23811_reviewer1.pdf [file pone.0251130.s004.pdf]
