# Supplementary material for: Aerial survey estimates of polar bears and their tracks in the Chukchi Sea
Source: PLoS One. 2021 May 6;16(5):e0251130. doi: 10.1371/journal.pone.0251130 (PMC8101751; doi:10.1371/journal.pone.0251130)
Supplement: Attachment — Submitted filename: Response to Reviewers2.docx [file pone.0251130.s006.docx]

**POINT-BY-POINT RESPONSES TO REVIEWER COMMENTS**

Here we provide point-by-point responses to all reviewer comments (our responses are in blue). We thank the reviewer for the time they took to examine our manuscript. We believe the changes we have made in response to these comments will clarify the modeling description and more accurately convey key uncertainties of our analysis.

Reviewer #2: This is an interesting attempt to estimate polar bear abundance in the Chukchi Sea. The major constraint on the study is the very small sample size. Only 8 groups were observed in US waters and 49 groups in Russian waters. By most standards, analyzing such a small sample would be of questionable merit given that 2 different platforms, different observation methods used, and varying conditions / habitats. I put little credibility in the actual results of abundance but the approach taken is rigorous (it’s unfortunate that more sightings weren’t made).

We agree with the reviewer that the sample size obtained in these surveys was not ideal for estimating animal abundance. Distance sampling, for instance, often suggests 60-80 encounters to be able to estimate the detection function accurately (see e.g. https://workshops.distancesampling.org/online-course/lecturepdfs/Ch4/L4-3%20Sample%20Size.pdf); our study used 49 for the Russian surveys, which is slightly under this recommendation. However, we do note that there are many examples in the published literature that use smaller samples to make inference about polar bear density from aerial surveys. For example, Evans et al. (2003) used a sample of 25 polar bear groups to estimate Chukchi-Beaufort polar bear density; McDonald et al. (1999) conducted inference using 15 polar groups; and Wiig and Derocher (1999) conducted inference using 29 bears. The low sample size is mostly attributable to the low densities of polar bears in these areas and are typical of springtime on-ice densities in much of the Arctic. In fact, our total survey effort (27,327 km) was greater than all 9 published studies we reviewed that use distance sampling to study polar bear abundance (Aars et al. 2009, 2017; Dyck et al. 2017; Evans et al. 2003; McDonald et al. 1999; Obbard et al. 2015; Stapleton et al. 2014, 2016; Wiig and Derocher 1999 – see S2 Appendix for citations), where the range of transects were all between 3,756 and 20,975 km. Our point is that while small, our sample sizes on the Russian side were comparable (and sometimes greater than) those in the peer-reviewed literature and that our study represents an unprecedented amount of survey effort compared to other polar bear surveys. The 49 groups counted on the Russian side allowed reasonable estimation of a detection function. The 8 groups observed on the US side were certainly not ideal and would be too few to estimate a detection function, but the additional 12 fly-over trials generated a reasonable estimate of detection probability that was independent of the main survey effort. The reviewer’s final point about varying conditions (specifically, sea ice melt) probably did impact the quality of our estimates near the Bering Strait where most melt occurred, but this was not an area where abundance was estimated to be high; in many areas (especially where abundance was estimated to be the highest), the quantity of sea ice was roughly constant during the period where surveys were conducted.

We made several changes and additions to the manuscript to ensure these comments are thoroughly addressed. First, we added text to the abstract and discussion emphasizing that the two survey platforms and other considerations meant that the methods used to generate abundance estimates depended on multiple assumptions:

“. . . a number of factors (e.g., equipment issues, differing platforms, low sample sizes, size of the study area relative to sampling effort) required us to make a number of assumptions to generate estimates . . .”

Regarding sample size, we added the following to the discussion:

“At the same time, it is important to note that bear densities, as well as sample sizes, will be reduced in the spring relative to late summer of fall surveys when the quantity of polar bear habitat (and therefore the size of potential study areas) is reduced (McDonald et al. 1999).” (Lines 529-532)

Finally, we included a new section in the discussion, “Additional limitations and potential impact on estimates” as a place to collect the many, varied caveats associated with our analysis, including sample size, changing habitat, and different platforms/observation methods.

My major concern with the study is the determination of the area to which the density estimates were applied: this is virtually a non-issue for the manuscript and totally ignored. The beige lines in Figure 1, if I’ve understood correctly, show the area to which the density estimates were applied. I suspect the large estimate is associated in part with applying the density to northern areas that may be almost unused at this time of year. I doubt the northern areas of the study area have a similar density to those nearshore but that is simply speculation (based on polar bear ecology and marine productivity) but the RSF may improve that fit although I found the description of how the RSF was applied cryptic. If the RSF is for only the period of the study, OK, that helps but I didn’t see much discussion on this point and all I know is that it’s inside the GAM.

There are two points here, one concerning possible extrapolations in the northern part of our study area, and one about the use of RSFs (and lack of a detailed description thereof) in the paper.

First, the reviewer is correct that there is potential for extrapolation bias in areas that are not surveyed. However, since our estimates are spatially explicit (see last row of Fig 4), one can calculate what abundance estimates are for certain areas in order to assess whether extrapolations should be regarded as problematic. For instance, taking a look for the estimate for the PBSG specialist group boundary (blue shading in Fig 1 that also overlaps with the beige grid), we get a time-averaged estimate of 3104 bears, compared to the estimate of the “full grid” of 3435 bears; stated another way, the abundance estimated in the unshaded area (primarily the northern and western portions of the study area, encompassing 32% of the study area) was estimated to be 331 bears. When looking at RSF plots which we now include in a new S1 Appendix, there appears to be predicted use in the far north of the study area. Therefore, we do not find evidence extrapolation is driving the magnitude of abundance estimates. Rather, it is the large number of encounters made in the southern half of the study area, particularly on the Russian side, that is driving the estimate.

Perhaps part of the confusion is that the maps of estimated abundance (Fig 4, bottom row) have a nonlinear color scaling such that predicted abundance has to get very close to zero to achieve a blue hue; this was a result of us wanting to show regions with low abundance (e.g. light blue) in addition to regions with high abundance (yellow). We have now included the following text in the figure caption, which should hopefully clarify this:

“Note that the scale of shading on abundance plots is nonlinear (i.e., very low densities are visible as light blue colors).”

Nevertheless, we agree with the reviewer’s broader point that our estimates are partially dependent on extrapolating to unsurveyed locations, which is potentially problematic if density-covariate relationships are unreliable in certain areas (e.g., the northern part of our study area). This is now addressed in the new subsection in the Discussion, entitled “Additional limitations and potential impact on estimates.”

Second, we agree with the reviewer that the RSF covariate development was cryptically described and that the manuscript would benefit from a fuller treatment. We have thus included a new appendix (S1 Appendix) that describes how the RSF was developed, and provides plots of predicted space use during our surveys. Briefly, RSFs were developed using data from multiple years of satellite telemetry records from adult female polar bears equipped with radiocollars. We then used the RSF, together with environmental covariate values from each day of our aerial surveys, to predict relative habitat utilization in each grid cell. As indicated in our cover letter, this revealed a data formatting error in how RSF distributions were entered into the original analysis. Previously, just 1 RSF surface was used instead of the full suite of daily RSF distributions. Correction of this error decreased abundance point estimates by about 3%. The plots in this appendix are useful for comparing RSF distributions to density predictions from our model, as well as to raw maps of polar bear sightings. In particular, it is evident that RSFs based on telemetered bears do not adequately predict the high densities of bears observed in Russian waters south of Wrangell Island. The low correlation between RSFs and our estimated density surface (ρ=0.25) suggests that the RSFs, which were based on dta from a sample of females that were collared near Kotzebue, Alaska may not accurately reflect spatial habitat use of the entire Chukchi subpopulation, at least in 2016. We have introduced additional text to the discussion to expand on this point.

I’m not overly convinced that tracks are a useful component – they are incredibly dependent on snow conditions and weather.

Given the susceptibility of tracks to environmental conditions and our findings that the track effect confidence interval considerably overlapped zero (line 488), we agree to a limited extent. However, we haven’t seen other studies that have tried to relate polar bear density to track density in the literature. If one could use track density to predict polar bear density it would be quite advantageous, since it is much easier to encounter tracks than it is bears themselves. As such, we treated it as a hypothesis worthy of investigation. To address the reviewer’s point and provide context to this, the discussion now states:

“Although further investigation with more consistent track sampling protocols would be worthwhile, the lack of support for a track density effect as a predictor of polar bear abundance suggests limited utility for population monitoring (i.e., as a relative abundance index). On the other hand, correlation between polar bear density estimates and track density was 0.63, higher than any other individual covariate.” (Lines 596-601)

We also indicate that

“. . . it is important to note that polar bear tracks indicate presence of bears at some point in time, but the longevity, visibility, and location of tracks was likely a function of many factors including sea-ice dynamics, surface characteristics (e.g., hard ice vs. deep snow), and weather.” (Lines 592-596)

On balance, I don’t really put much credence in the numbers produced but the authors have made the best of weak data and try to put the incredibly expensive data to use. As such, the study warrants presentation in a peer-reviewed journal so that others can learn of the various problems of conducting such a survey.

The reviewer is certainly correct that the data obtained during this study were imperfect, especially with equipment issues on the Russian side. We tried to do our best given the data available and are glad the reviewer sees value in this. We also agree with the reviewer that an important aspect of our paper is to communicate the difficulties encountered with our approach, such that method might be improved in the future. Given the difficulty and expense of studying ice seals and polar bears, and the fact that diminishing sea ice is making live-capture studies for polar bears more difficult, we anticipate that aerial survey methods for these species will see increased application in the future.


I think, however, a more cautious statement of caveats would be useful. Most of the major limitations of the study (e.g., sample size, extrapolation of RSFs, tracks, area of application) are glossed over.

We think this is a good suggestion and appreciate the reviewer’s attention to these important details. As mentioned earlier, we now devote a new subsection of the discussion to ``Additional limitations and potential impact on estimates” where we talk more specifically about sample size, extrapolation, and area of application. We also allude to these issues in the abstract now, indicating that

“. . . a number of factors (e.g., equipment issues, differing platforms, low sample sizes, size of the study area relative to sampling effort) required us to make a number of assumptions to generate estimates . . .”

Abstract (no line #)
“are larger than” – I suggest adding “point estimates” – there is no statistical difference between the earlier and current estimates (although it’s almost impossible to have a difference given the large confidence intervals

Changed

it’s unclear why the lower bound is considered useful but I’m OK with leaving it in – I don’t believe, however, that it is very useful as everyone will use the point estimates

We believe that the credible interval of the abundance estimate using g(0) = 1 does indeed provide useful information on the likely lower bound of abundance in this region, and so have retained the original text.

2 - binomial name

Added

5 – what does “demographic status” mean? This is an odd bit of wording.

We agree that this was unclear. Changed to “population trends”

17-8 – superscripts missing

Fixed

106 & 113 – SI units (km/h) for speed

Changed

366 – it’s to its

Fixed

385 – it is unusual to have groups as the primary sampling unit. Subadults, adult males, and solitary females would make up >50% of the population.

Using groups as the sampling unit is standard in aerial survey / distance sampling literature. The reason is that detections are made of groups of bears rather than individual bears independently. Assuming that individual bears are detected independently would inflate sample size and artificially increase precision. As such, detections are often modeled at the group (or “cluster”) level and then a separate model for group size is used to expand to the total number of individuals in the population. We now cite Buckland et al. (2001) as support for this procedure. (line 386)

499-400 “Translation of polar bear abundance to density is somewhat complicated by the fact that sea ice changed considerably throughout the survey” This is only one aspect. It is extremely likely that the density estimate does not apply over the whole area shown in Figure 1 (beige grid). That the density along the coast was similar to the north areas does not fit well for what is known about polar bears. Relying on Regehr et al. 2018 is of questionable merit as there is little basis for extrapolating those estimates to the whole population. In essence, a major peril in density extrapolation to obtain a population estimate is knowing how density varies over the area. Without such insight, the population could be increased or decreased to any size based on the area to which density is applied. The RSF may assist as a proxy for density and the results are unclear on how much influence this had on the population estimate.

The caveat of area and density variation over the study area is pretty much a non-issue in the manuscript. This needs attention. The lack of context for the density results appears to be a major oversight. Comparing only to a single paper in the same area is questionable.

Please see previous responses to “general comments.” In particular, we think the reviewer may have been misinterpreting our density plots (Final row of Fig 4.) which show how estimated density varies over the landscape. These estimated densities are a direct result of the GAM-like modeling framework where polar bear counts are related to environmental covariates, and these covariates are used to predict a density surface of bears in the study area (which varies spatially as well as temporally). Nevertheless, we provide additional context and collect caveats of the estimation process in a new section, “Additional limitations and potential impact on estimates”

It is difficult to provide many other comparisons of density in the study area because of the general lack of studies that have quantified density in this region, particularly in Russian waters. However, we now make comparisons to two pilot aerial surveys conducted in part over the U.S. portion of the Chukchi Sea (Evans et al. 2003; McDonald et al. 1999) – see lines 564-568. We also provide additional text in the Discussion comparing RSF distributions to our estimated density surfaces (Lines 602-610).
